# A clathrin mediated endocytosis scaffolding protein, Intersectin 1, changes in an isoform, brain region, and sex specific manner in Alzheimer’s disease

**DOI:** 10.3389/fnins.2024.1426180

**Published:** 2024-06-10

**Authors:** Sierra Jaye, Ursula S. Sandau, Trevor J. McFarland, Randy L. Woltjer, Julie A. Saugstad

**Affiliations:** ^1^Department of Anesthesiology and Perioperative Medicine, Oregon Health and Science University, Portland, OR, United States; ^2^Division of Neuropathology, Department of Pathology, Oregon Health and Science University, Portland, OR, United States

**Keywords:** Intersectin 1, Alzheimer’s disease, clathrin mediated endocytosis, human brain, 5xFAD

## Abstract

Alzheimer’s disease (AD) is the most common form of dementia and is characterized by the accumulation of amyloid-beta (Aβ) plaques and neurofibrillary Tau tangles in the brain. We previously identified a set of candidate AD microRNAs (miRNAs) in human cerebrospinal fluid (CSF) and used a target prediction pipeline to identify mRNAs and pathways that could potentially be regulated by the miRNAs. Of these pathways, clathrin mediated endocytosis (CME) was selected for further investigation. CME is altered in multiple brain cell types in AD and is implicated in early cellular phenotypes such as enlarged early endosomes and pathogenic processing of Aβ. However, a comprehensive evaluation of major CME hub proteins in humans with AD across multiple brain regions is lacking. Thus, we used immunoblots to evaluate human post-mortem AD and control (CTL) frontal cortex (FC; AD *n* = 22, CTL *n* = 23) and hippocampus (HP; AD *n* = 34, CTL *n* = 22) for changes in Intersectin 1 (ITSN1), Phosphatidylinositol Binding Clathrin Assembly Protein gene (PICALM), Clathrin Light Chain (CLT), FCH and Mu Domain Containing Endocytic Adaptor 1 (FCHO1), Adaptor Related Protein Complex 2 (AP2) Subunit Alpha 1 (AP2A1), and Dynamin 2 (DNM2). Of these, we found that in AD, ITSN1-long (ITSN1-L) was decreased in the FC of males and HP of females, while ITSN1-short was increased in the HP of both males and females. We further evaluated ITSN1-L levels in cortex (CTX) and HP of the 5xFAD mouse model of Aβ pathology at different timepoints during aging and disease progression by immunoblot (*n* = 5–8 per group). At 3 months, female 5xFAD exhibited an increase of ITSN1-L in CTX but a decrease at 6 and 9 months. Additionally, immunofluorescent staining of 5xFAD primary HP neurons showed an increase of ITSN1-L in matured 5xFAD neurons at 21 and 28 days *in vitro*. Together, our studies show that in AD, isoforms of ITSN1 change in a brain region-and sex-dependent manner. Further, changes in ITSN1-L are transient with levels increasing during early Aβ accumulation and decreasing during later progression. These findings suggest that ITSN1 expression, and consequently CME activity, may change depending on the stage of disease progression.

## 1 Introduction

Alzheimer’s Disease (AD) is characterized by the accumulation of amyloid beta (Aβ) plaques and neurofibrillary Tau protein tangles as the disease progresses (Braak and Braak, 1991; Selkoe and Hardy, 2016). However, as there are no reliable tools to detect early brain changes in AD, patients are often diagnosed 15–20 years after the onset of neuropathology (Deture and Dickson, 2019; Alzheimer’s Association, 2023). Many scientists and clinicians are now focused on the development of more sensitive, affordable, and less invasive diagnostic tools, as well as disease modifying treatments to halt disease progression. To that end, we previously discovered (Lusardi et al., 2016) and validated (Wiedrick et al., 2019) a set of candidate microRNA (miRNA) biomarkers in human cerebrospinal fluid (CSF) that distinguish AD patients from neurologically normal controls (CTL).

MiRNAs regulate protein expression via complementary binding to targeted messenger RNA (mRNA) sequences. This leads to suppression or degradation of the targeted mRNA transcripts and subsequently reduced translation of the corresponding protein (Filipowicz et al., 2008; Shyu et al., 2008; Gebert and Macrae, 2019). As miRNAs themselves are also highly regulated, factors that alter their expression, such as disease state, can affect target mRNA protein translation (Gebert and Macrae, 2019). We previously published a target prediction analysis in which we utilized these novel candidate miRNA biomarkers to uncover potential cellular pathways involved in AD (Sandau et al., 2020b). We found that a significant number of proteins involved in clathrin mediated endocytosis (CME) may be regulated by AD CSF miRNAs.

CME is a ubiquitous process by which cells internalize material from the cell surface and mediates many cellular functions (Doherty and McMahon, 2009; Kaksonen and Roux, 2018). As such, disruption of CME function is associated with many diseases (McMahon and Boucrot, 2011; Schreij et al., 2016). In humans with AD, genetic links have been made to Adaptor Related Protein Complex 2 (*AP2*) and Phosphatidylinositol Binding Clathrin Assembly Protein (*PICALM*) (Raj et al., 2018; Azarnia et al., 2019) and many proteins involved in CME have reported changes at the RNA and protein level over a wide variety of brain regions (Yao et al., 1999, 2000, 2003; Cao et al., 2010; Jaye et al., 2024). There are many cellular functions disrupted in AD that are downstream of CME or use CME machinery. For example, one of the earliest pathologies specific to sporadic AD is the enlargement of early endosomes in pyramidal cells (Cataldo et al., 1997, 2000; Nixon, 2005). This in turn results in endolysosomal disruption (Nixon, 2017; Colacurcio et al., 2018; Szabo et al., 2022) and various neuropathological outcomes such as Aβ_1–42_ over production (Koo and Squazzo, 1994; Wu and Yao, 2009; Burrinha et al., 2021) and clearance deficits (Narayan et al., 2020) as well as dystrophic neurites (Sanchez-Mico et al., 2021). Increased size and/or number of endosomes correlates with increased endocytosis (Stenmark et al., 1994) and suggests that CME changes may contribute to downstream endolysosomal changes in AD. Inhibition of CME both reduces internalization of Amyloid Beta Precursor Protein (APP) (Burrinha et al., 2021) and prevents Aβ induced axon damage (Kuboyama et al., 2015).

Synapse dysfunction is another well characterized neuronal phenotype in AD (Pei et al., 2020), which correlates with cognitive impairment and leads to severe synaptic loss in early stages of disease (Terry et al., 1991; Jacobs and Kahana, 2010). One facet of synapse dysfunction in AD is the reduction of pre-synaptic vesicle recycling, which normally relies on CME to maintain a readily available pool of vesicles for fast transmission (Cirrito et al., 2008; Pechstein et al., 2010; Milosevic, 2018). Blocking CME significantly reduces vesicle recycling in pre-synaptic terminals (Doherty and McMahon, 2009). Additionally, post-synaptic CME may contribute to AMPA receptor trafficking, which contributes to cognitive function (Man et al., 2000; Lacor et al., 2004; Snyder et al., 2005; Guntupalli et al., 2017). This together suggests that alterations of CME could contribute to both pre-and post-synaptic dysfunction in AD.

The current study examines how the expression of proteins in the CME pathway may be altered in human postmortem AD brains, the 5xFAD mouse model of amyloidosis, and 5xFAD primary neurons. Here, we examined, in both males and females, levels of CME proteins that have been previously implicated in human AD: Intersectin 1 (ITSN1) (Wilmot et al., 2008; Malakooti, 2019), Clathrin Light Chain A (CLTA) (Nakamura et al., 1994), PICALM (Raj et al., 2018; Azarnia et al., 2019; Alsaqati et al., 2023), FCH and Mu Domain Containing Endocytic Adaptor 1 (FCHO1) (Teerlink et al., 2021), Adaptor Related Protein Complex 2 (AP2) Subunit Alpha 1 (AP2A1) (Yao et al., 2000; Srinivasan et al., 2022), and Dynamin 2 (DNM2) (Aidaralieva et al., 2008; Kamagata et al., 2009; Du et al., 2021). We found that ITSN1 is altered in human AD brains compared to age-matched CTLs in an isoform-, brain region-, and sex-specific manner. These findings were also repeated in aged, 9 month old 5xFAD mice, a timepoint associated with Aβ pathology, synaptic loss and cognitive impairment (Forner et al., 2021; Oblak et al., 2021). Further, using 1, 3, 6, and 9 months old 5xFAD and wildtype (WT) mice, we demonstrate a transient increase in ITSN1-L in female 5xFAD mice early in disease when Aβ deposits begin accumulating, that later decrease to below CTL levels by 6 months of age. This, and our finding that ITSN1-L increases in 5xFAD primary HP neurons, illustrate a potential correlation of ITSN1-L to increase with developing Aβ pathology. These data further implicate a role for CME in AD progression given that both the neuronal specific ITSN1-L, and the ubiquitous ITSN1-S, interact with many essential CME proteins (McMahon and Boucrot, 2011; Wong et al., 2012; Gubar et al., 2013; Herrero-Garcia and O'Bryan, 2017), and that overexpression (Sengar, 1999; Simpson et al., 1999) and under-expression (Yu et al., 2008; Thomas et al., 2009) of ITSN1 significantly reduces CME. Interestingly, ITSN1-L has several additional neuronal specific functions (Tsyba et al., 2008, 2011) including dendritic spine development (Irie and Yamaguchi, 2002), fast neurotransmission (Sakaba et al., 2013; Jäpel et al., 2020), synaptic plasticity through reelin (Jakob et al., 2017), and mitogenic signaling pathways (O’Bryan et al., 2001). Further, ITSN1-L knockout mice have learning and memory deficits (Malakooti et al., 2020). Thus, the differences we show in ITSN1-L levels between brain region and disease stage also point to the potential of ITSN1-L affecting multiple aspects of AD progression.

## 2 Methods

### 2.1 Human brain samples

This study consisted of post-mortem tissue of 101 patient donors to the Oregon Health & Science University (OHSU), Layton Aging and Alzheimer Disease Center Brain Bank. All donors were aged individuals from the community and had either no known neurological disease (CTL) or a clinical history of AD established, as previously described (Tierney et al., 1988; Nelson et al., 2014). After consent was given from the next of kin, brain autopsy was performed on all donors in accordance with OHSU guidelines and ethical approval for this work. Tissue was stored unfixed at −80°C until use.

### 2.2 Animals

All animal experiments were approved by the OHSU Institutional Animal Care and Use Committee (TR01_IP00000040) and were conducted in compliance with relevant ethical regulations for animal research. We purchased 5xFAD mice from JAX (The Jackson Laboratory, #034840-JAX). These mice contain three familial AD mutations in humanized *APP* (K670N/M671L, 1716 V, V717I) and two familial AD mutations in humanized *PSEN1* (M146L, L286V). Male 5xFAD hemizygous were crossed with female B6SJLF1/J (JAX Strain #:100012). Animals were housed in standard conditions of temperature, light, and enrichment, and provided food and water *ad libitum*. After genotyping and weaning at 21 days, mice were social housed with littermates and aged until the correct timepoint reached. Some male mice developed aggression after 4 months of age and were subsequently housed individually.

### 2.3 Genotyping

For genotyping, primers that detect the mutant and wild-type (WT) APP amplicon (Forward: 5’-ACCCCCATGTCAGAGTTCCT-3′, mutant reverse: 5’-CGGGCCTCTTCGCTATTAC-3′, and WT reverse: 5’-TATACAACCTTGGGGGATGG-3′) were used to determine presence of the transgenes. DNA extraction and PCR on tail snips (collected from weanlings or embryos) was performed with the REDExtract-N-Amp Tissue PCR Kit (Sigma Aldrich) and bands resolved on an agarose gel. The touchdown PCR thermal cycling protocol used is as follows: initial denaturation (95°C, 3 min, 1 cycle), denaturation (94°C, 30 s), then 10 1-min cycles starting at 65°C and decreasing by 0.5°C each cycle (65–61°C) with extension at 68°C for 1 min after each, then 28 cycles of 95°C for 30 s, 60°C for 1 min, and 72°C for 1 min, after these cycles is 72°C for 2 min.

### 2.4 Animal tissue collection

Mice were euthanized at 1, 3, 6, and 9 months via CO_2_ inhalation, followed by cervical dislocation. Brains were removed and hemispheres separated along the midline. The left hemisphere was dissected to collect the HP and CTX, which were frozen on dry ice and stored at −80°C until immunoblot analysis. The right hemisphere was immersed and fixed in 4% paraformaldehyde (PFA) overnight then transferred to 30% sucrose in PBS for 2 days, all at 4°C. The right hemisphere was then frozen over dry ice and sectioned coronally at 30 μm using a cryostat (Cryostar NX50, Epredia). Serial sections were collected into 12-well plates containing cryoprotectant (30% ethylene glycol, 20% glycerol, 50%PBS) and stored at 4°C for subsequent free-floating tissue staining.

### 2.5 Immunoblot

For human brain samples, archived frozen brain tissue was thawed, and while thawing the white matter and leptomeninges were dissected away. Forty to sixty milligram of post-mortem gray matter from AD and CTL frontal cortex (FC; AD *n* = 22, CTL *n* = 23) and HP (AD *n* = 34, CTL *n* = 22) was collected, and total protein extracted by sonication for 25 s in lysis buffer (62.5 mM Tris pH 6.8, 2% SDS and 10% glycerol) followed by centrifugation at 14,000 g for 10 min. The supernatant was aliquoted and stored at −80°C until used for immunoblot analysis. Before use, protein lysates were analyzed for protein content using the Qubit Protein Assay kit (ThermoFisher Scientific (TFS)), loading buffer (50 mM DTT, 0.01% bromophenol blue) added, boiled, then cooled before protein (50 μg: ITSN1, 40 μg: AP2A1, FCHO1, 20 μg: CLT, DNM2, PICALM) was loaded onto precast NuPAGE 3–8% Tris-Acetate gels (Invitrogen) and separated by electrophoresis.

Fresh frozen mouse tissue, collected as described above, was thawed and incubated in RIPA lysis buffer (TFS) with Halt protease/phosphatase inhibitor. Tissue was homogenized with a handheld mechanical pestle for 10 s, then digested in the RIPA buffer for 30 min, vortexing halfway through. After protein extraction, samples were centrifuged at 12,000 g for 20 min at 4°C. Supernatant was collected and aliquoted for BCA analysis (Pierce BCA Protein Assay kit, TFS) and immunoblot. Laemmli running buffer was added to samples, which were then boiled, cooled, and stored at −80°C until 10 μg of protein was loaded onto pre-cast NuPAGE 3–8% Tris-Acetate gels (Invitrogen) and separated by electrophoresis.

For both human and mouse gels after sample electrophoresis, protein was transferred onto Immobilon-FL polyvinylidene difluoride (PVDF) membranes (Millipore) in NuPAGE transfer buffer (Invitrogen) containing 10% methanol for better transfer of high molecular weight proteins. Before primary antibody incubation, membranes were dried overnight at 4°C then activated with methanol and stained with Revert 700 Total Protein Stain Kit Total protein stain (TPS, LI-COR Biosciences), imaged on the Odyssey CLx Imager (LI-COR Biosciences), then destained and blocked with Intercept (TBS) Blocking Buffer (LI-COR Biosciences). After blocking, membranes were incubated with the specified primary antibodies (Supplementary Table S1) for 2 h at room temperature in Intercept blocking buffer (LI-COR Biosciences) with 0.2% Tween-20. After washing (4 × 5 min, TBS-T, 0.2% Tween-20), membranes were incubated with the appropriate IRDye secondary antibodies (LI-COR, Supplementary Table S1) in Intercept blocking buffer with 0.2% Tween-20 and 0.02% SDS for 1 h at room temperature. Membranes were washed (4 × 5 min, TBS-T) and bands visualized on the Odyssey CLx Imager. For human samples with low abundance proteins that had high signal to noise ratios with the standard method, such as ITSN1, we used the iBind Flex automated western system (TFS), which has been shown to increase sensitivity (Sormunen et al., 2023). The intensity of each signal was determined with Empiria Studio software version 2.2 (LI-COR Biosciences) using the Quantitative Western Blot function that employs automatic lane detection and background subtraction to reduce variability of between gel analyses. Signals were first normalized within gels to TPS, and as multiple gels were needed to include all samples, bands were normalized to a repeated sample run on every gel to account for between gel variation.

### 2.6 Primary neuronal cultures

Primary HP neurons were collected, frozen as in Ishizuka and Bramham (2020), and grown in sandwich culture with glia as in Kaech and Banker (2006) to enable lower density culture. Briefly, both male and female at embryonic day (E) 15 were collected from timed pregnant 5xFAD hemizygous mice, and the HP dissected from the brain. To determine the embryo genotypes, PCR with embryo tails was run simultaneously with dissection. After genotypes were confirmed, HP of each genotype were pooled and incubated in 0.25% trypsin at 37°C for 15 min. Cells were then gently homogenized with a flame polished glass pipet into single cell suspension and counted. 500,000 cells were then added to cryovials containing freezing media (80% fetal bovine serum, 20% DMSO), frozen and stored in liquid nitrogen.

To prepare sandwich culture, 18 mm round coverslips were cleaned in 70% nitric acid for 48 h, sterilized by glass autoclave, and then had wax feet applied. They were then coated with 1 mg/mL poly-l-lysine overnight at room temperature and washed with PBS the next day. One week ahead of neuronal plating, 50,000 mouse neural stem cells were seeded into Primaria 60 mm cell culture dishes (Corning, 353,802) in differentiation media (10% fetal bovine serum, 10% 10X N2 supplement (Kaech and Banker, 2006), 100 U/mL penicillin–streptomycin) and new media added every other day, these become glial feeder cells. Twenty four hours before neuronal plating, glial feeder medium is replaced with neuronal media (B27 Plus Neuronal culture system, TFS).

Frozen neurons were thawed gently in a 37°C water bath for 2 min. In a separate 60 mm dish from glial feeders, neurons were plated at 150,000 cells/dish on the prepared side of the coverslips in neuronal plating media (0.6% wt/vol D-Glucose, 10% fetal bovine serum in minimum essential medium). After 3–4 h, when neurons have attached, the coverslips were flipped into dishes with glial feeder cells and the neurons grown on the underside of the coverslip held above the glial cells by wax feet, enabling trophic support from the glia. Once established, cultures were fed with neuronal medium once per week and coverslips collected and fixed with 10% neutral buffered formalin for 30 min at room temperature at 7, 14, 21, and 28 days *in vitro* (DIV). Coverslips were dehydrated in a gradient of ethanol (50–70–100%, 1 min each) and stored in 100% EtOH at −20°C until stained. For each time-point, *n* = 2–3 separate primary culture preparations were used with three coverslips collected from each culture preparation at each timepoint for a total *n* = 39–45 neurons. Within each time-point all coverslips were stained together for analysis.

### 2.7 Immunofluorescence

Mouse brain tissue sections were removed from cryoprotectant and washed three times in PBS then blocked with 10% normal goat serum in PBS for 2 h at room temperature. Rabbit anti-ITSN1 (1:500, Novus Biologicals) and rat anti-alpha tubulin (1:500, TFS) primary antibodies were applied overnight at 4°C in 10% normal goat serum. After washing in PBS, Alexa Fluor 488 Goat anti-Rabbit IgG (1:500, TFS) and Alexa Fluor 647 Goat anti-Rat IgG (1:1,000, TFS) secondary antibodies in 10% normal goal serum were incubated for 2 h at room temperature and counterstained with 4′,6-diamidino-2-phenylindole (DAPI). Sections were then mounted onto slides and dried before applying coverglass with ProLong Gold antifade mountant (TFS).

Fixed primary HP neurons were rehydrated by incubation for 1 min each in 70% ethanol, 50% ethanol, and then PBS. They were then permeabilized in PBS-T (0.1% Tween-20) for 10 min at room temperature then blocked for 30 min. Rabbit anti-ITSN1 (1:500, Novus Biological) and chicken anti-MAP2 (microtubule associated protein 2, 1:10,000, TFS) primary antibodies were applied overnight at 4°C. After washing with PBS, Alexa Fluor 647 Donkey anti-Chicken IgY (1:500, TFS) and Alexa Fluor 568 Donkey anti-Rabbit IgG (1:500, TFS) secondary antibodies were incubated for 1 h at room temperature and counterstained with DAPI. Blocking, primary, and secondary antibody incubation were all in 3% bovine serum albumin. Coverslips were mounted on glass slides with ProLong Gold antifade mountant (TFS). Specific antibody information is listed in Supplementary Table S1.

### 2.8 Image acquisition and analysis

Stained mouse hemispheres, 3 animals from each group (timepoint × sex) and 4 sections per animal, were fluorescently imaged over multiple z planes via Zeiss Axioscan 7 Slidescanner and a Plan-Apochromat 10X/0.45 M27 objective. Images were stitched together, and max projections created for comparison of staining across groups. All images within a time-point are displayed with the same settings.

Primary HP neurons were imaged using confocal laser scanning microscopy using an LSM 900 Axio Observer.Z1/7 (Zeiss) inverted microscope equipped with a Plan-Apochromat 63x/1.40 NA oil immersion objective, 1.00 Airy Units/56 μm pinhole for each channel. Five to ten cells were imaged in three dimensions from each coverslip for total *n* = 39–45 neurons for each genotype at each timepoint. For analysis, maximum projections of each image were created from 5 z steps around the focus plane with sharpest signal. Signal intensities of maximum projections were collected using the Intellesis automated segmentation package in Zeiss ZEN 3.6. Intellesis segmentation is a machine learning segmentation tool in which a model is trained to segment image objects in complex images such as neurons. We trained the program to segment neurons based on MAP2 signal then collected the signal intensity of the ITSN1 staining within the MAP2 segmentation to analyze.

### 2.9 Statistical analysis

For both the immunoblot and immunofluorescence experiments, the experimenter was blinded to sample demographics or mouse genotype until all data were collected. Data were analyzed with GraphPad Prism software v10.1.0 (GraphPad Software, Inc., San Diego, CA). To assess differences between protein levels in human AD versus CTL or 5xFAD and WT mice while considering sex (male versus female) we used a two-way ANOVAs (genotype × sex) followed by Šídák multiple comparisons tests, when appropriate. A *p* < 0.05 was considered to be a significant difference between groups. Data were not compared between time-points for either immunoblot or primary neuron experiments as samples of each time-point were prepared and assayed at different times.

For immunoblots using post-mortem human brain, all HP samples were run together and probed for antibody together, while all FC samples were grouped in the same way for each protein. Prior to running the immunoblot gels all protein lysates within each brain region were randomized for disease and sex. Immunoblot data are shown as mean protein levels ± the standard error of the mean (SEM) normalized to TPS versus experimental groups in both the FC and HP: CTL-male (FC *n* = 12; HP *n* = 11), AD-male (FC *n* = 10; HP *n* = 17), CTL-female (FC *n* = 11; HP *n* = 11), AD-female (FC *n* = 12; HP *n* = 17). Biological replicates are displayed as individual symbols on each graph. Outliers were identified using ROUT analysis with Q = 1% (Motulsky and Brown, 2006).

Mouse immunoblot data are shown as mean ± SEM of ITSN1 protein levels normalized to TPS within each age group for both the CTX and HP of male and female 5xFAD and littermate WT mice. Immunoblots for each timepoint were run together. Biological replicates are displayed as individual symbols on each graph. Outliers were identified using Grubb’s test with α = 0.05.

Data from primary neurons are shown as mean signal intensity of ITSN1 within the MAP2 segmentation for each genotype at 7, 14, 21, and 28 DIV. Experiments were carried out independently 2–3 times per group and imaged across three coverslips per repetition for a total *n* = 39–45 cells per group. To assess differences in ITSN1 signal intensity between 5xFAD and WT neurons we used unpaired, two-tailed, Student’s *t*-tests at 7, 21, and 28 DIV, and Welch’s corrected *t*-test at 14 DIV.

## 3 Results

### 3.1 The CME pathway contains predicted targets of AD CSF miRNAs

Our previous study evaluated potential targets of 5 trending AD CSF miRNAs (miRs-142-3p, 146a-5p, 146b-5p, 193a-5p, 365a-3p) (Wiedrick et al., 2019; Sandau et al., 2020b). This initial analysis consisted of miRNA target prediction and subsequent pathway analysis. Many of these pathways were already known to be related to or altered in AD (Sandau et al., 2020a). Notably, CME emerged as a pathway of interest that is predicted to be targeted by the AD CSF miRNAs. Table 1 lists proteins involved in CME whose mRNA are predicted targets of AD CSF miRNAs. Of these, we evaluated ITSN1, CLTA, and PICALM for changes in expression between male and female CTL and AD brain. We also quantified FCHO1, AP2A1, and DNM2 as these are integral to CME and implicated as disrupted in AD (Nakamura et al., 1994; Yao et al., 2000; Wilmot et al., 2008; Malakooti, 2019; Du et al., 2021; Teerlink et al., 2021; Srinivasan et al., 2022), but are not predicted targets of the AD CSF miRNAs.

**Table 1:** Proteins involved in CME are targeted by one or more candidate AD CSF MiRNA biomarkers.

mRNA target symbol	MiRNA (hsa-miR)	mRNA target name
*CHP1*	365a-3p	Calcineurin Like EF-Hand Protein 1
**CLTA**	142-3p	Clathrin Light Chain A
*CTTN*	142-3p	Cortactin
*EPN1*	142-3p	Epsin 1
*FGF1*	365a-3p	Fibroblast Growth Factor 1
*FGF9*	142-3p	Fibroblast Growth Factor 9
*HGS*	142-3p	Hepatocyte Growth Factor-Regulated Tyrosine Kinase Substrate
*HSPA8*	365a-3p	Heat Shock Protein Family A (Hsp70) Member 8
*ITGB8*	142-3p	Integrin Subunit Beta 8
**ITSN1**	193a-5p	Intersectin 1
*NUMB*	146a-5p	NUMB Endocytic Adaptor Protein
	146b-5p	
**PICALM**	142-3p	Phosphatidylinositol Binding Clathrin Assembly Protein
*PIK3R3*	365a-3p	Phosphoinositide-3-Kinase Regulatory Subunit 3
*PIK3R6*	142-3p	Phosphoinositide-3-Kinase Regulatory Subunit 6
*PPP3R1*	142-3p	Protein Phosphatase 3 Regulatory Subunit B, Alpha
*RAC1*	142-3p	Rac Family Small GTPase 1
	146a-5p	
	146b-5p	
	365a-3p	
*STAM*	142-3p	Signal Transducing Adaptor Molecule
*SYNJ1*	365a-3p	Synaptojanin 1
*VEGFB*	193a-5p	Vascular Endothelial Growth Factor B
*WASL*	142-3p	WASP Like Actin Nucleation Promoting Factor

The predicted mRNA targets of candidate AD miRNAs that are involved in CME. Target mRNA symbol, miRNAs predicted to regulate the target, and target name are shown in alphabetical order. Targets we performed immunoblots on are indicated in bold.

### 3.2 Donor demographics for post-mortem brain tissues

Specific demographics for the postmortem brain samples from AD and CTL donors obtained from the OHSU Layton Aging and Alzheimer’s disease research center are in Table 2. The samples included 45 fresh frozen samples from the FC (23 CTL, 22 AD) and 56 samples from the HP (22 CTL, 34 AD) for a total of 101 samples used for immunoblot in four groups (disease by sex). Disease state of all samples used were confirmed by post-mortem assessment with CTLs being Braak stage I/II and AD being Braak V/VI. There was no significant difference in the number of males to females, or age, at time of death between groups. All samples had postmortem intervals of less than 24 h but given the constraints in availability of tissue there was a significant difference of postmortem interval between male AD and CTL in both the FC (*p* = 0.02) and HP (*p* = 0.005).

**Table 2:** Participant demographics for immunoblots.

	Frontal cortex		Hippocampus	
	CTL	AD	CTL	AD
Sex				
Female	11	12	11	17
Male	12	10	11	17
Total	23	22	22	34
Age (Mean ± SD)				
Female	70 ± 10	76 ± 6	71 ± 11	74 ± 9
Male	68 ± 9	76 ± 5	70 ± 10	74 ± 8
Total	69 ± 9	76 ± 6	70 ± 10	74 ± 8
PMI (Mean ± SD)				
Female	11 ± 7	14 ± 7	13 ± 8	9 ± 7
Male	17 ± 6	10 ± 6	19 ± 3	11 ± 7
Total	14 ± 7	12 ± 7	16 ± 6	10 ± 7

Sex, mean age ± standard deviation (SD), and mean postmortem interval (PMI) ± SD of participant samples used for immunoblots. The sex, age, and PMI were matched across groups to the best of our ability in order to mitigate these factors as potential confounds.

### 3.3 ITSN1 is decreased in AD FC and HP human postmortem brain

**Figure 1:**
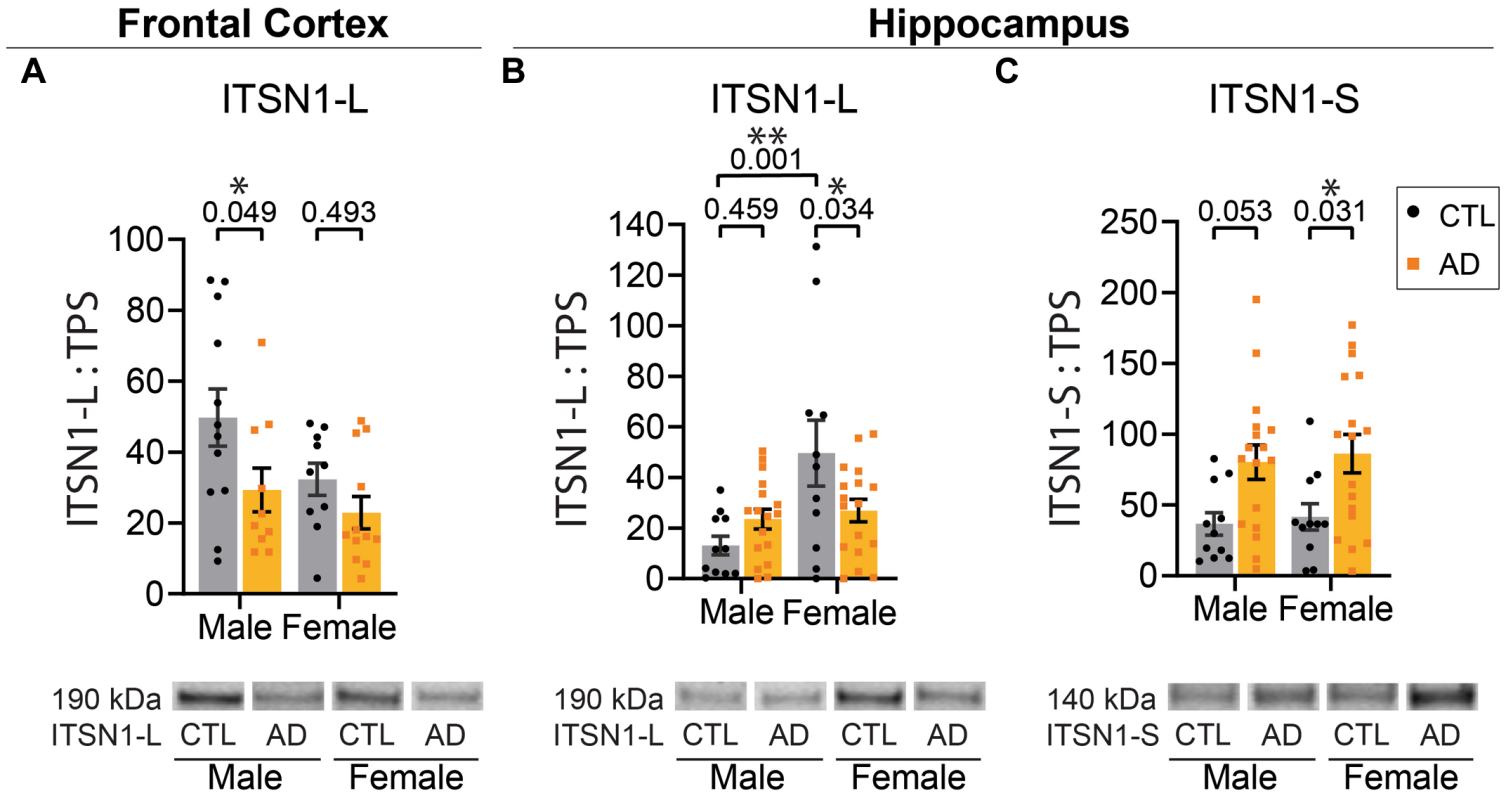
ITSN1-L and ITSN1-S are altered in human AD brain. **(A)** Comparison of ITSN1-L in the FC of male and female AD and CTL protein lysates. **(B)** Comparison of ITSN1-L and **(C)** ITSN1-S in the HP of male and female AD and CTL protein lysates. For each analysis, all graphs **(A–C)** show protein intensity values normalized to total protein stain (TPS) with representative immunoblot bands underneath. Data are represented as mean ± SEM. Statistical significance was determined by two-way ANOVA, with *post hoc* Šídák multiple comparisons tests for analyses with significant group effects. *p < 0.05; **p < 0.01.

We first investigated ITSN1 expression, as it interacts with many hub proteins in CME (Oughtred et al., 2021). There are two isoforms resulting from alternative splicing (Hussain et al., 1999; Okamoto et al., 1999): a neuronal-specific long form (ITSN1-L) and a ubiquitous short form (ITSN1-S). The ITSN1 antibody is expected to detect both ITSN1-L (190 kDa) and ITSN1-S (140 kDa) isoforms. In the FC, there was a significant main effect of AD on ITSN1-L levels (*p* = 0.021) (Figure 1A). Post-hoc multiple comparisons tests showed a significant decrease of ITSN1-L in AD males as compared to CTL males (*p* = 0.049), but no difference between AD and CTL females (*p* = 0.493). FC ITSN1-S signal was very faint and quantification attempts resulted in high signal to noise ratios, thus ITSN1-S levels were not evaluated in the FC.

In the HP, there was a main effect of sex on ITSN1-L levels (*p* = 0.004) and a significant interaction between disease and sex (*p* = 0.014) (Figure 1B). Post-hoc tests demonstrated that in AD females there was a significant decrease in ITSN1-L, as compared to CTL females (*p* = 0.034), but no difference between CTL and AD males (*p* = 0.459). We also found that in CTL males ITSN1-L is significantly decreased, as compared to CTL females (*p* = 0.001). Also in the HP, ITSN1-S levels showed a significant main effect of disease (*p* = 0.001), with significant increases in female AD versus CTL (*p* = 0.031) and a trending increase in male AD versus CTL (*p* = 0.053) (Figure 1C). All statistics are shown for the FC in Supplementary Table S2 and HP in Supplementary Table S3.

### 3.4 Other CME proteins are not altered by AD in the human brain

We examined other proteins also involved in the CME pathway, including CLTA, CLTB, PICALM, FCHO1, AP2A1, and DNM2 in the FC and HP of AD and CTL humans by immunoblot. In the FC, CLTA and CLTB showed no change in AD, but revealed a significant sex difference in CTLs (Supplementary Figures S3A,B). Previously, PICALM was shown to have differences in AD between isoforms (Ando et al., 2013, 2016; Alsaqati et al., 2023), so we evaluated the effect of AD on both isoforms 1,2 (65–75 kDa), and isoform 4 (50 kDa). In our sample cohort, PICALM isoforms 1,2 showed no significant effect of disease, sex, or an interaction in either the FC (Supplementary Figure S1A) or HP (Supplementary Figure S2A). Likewise, evaluation of PICALM isoform 4 showed no significant effect of disease, sex, or an interaction in the FC (Supplementary Figure S1B), but in the HP there was a trending disease by sex interaction (*p* = 0.051, Supplementary Figure S2B). FCHO1, AP2A1, and DNM2 levels were not significantly affected by disease, sex, or their interaction in the FC (Supplementary Figures S1C–E) or HP (Supplementary Figures S2C–E). All statistics are shown for the FC in Supplementary Table S2 and HP in Supplementary Table S3.

### 3.5 ITSN1 shows widespread staining in WT mice

**Figure 2:**
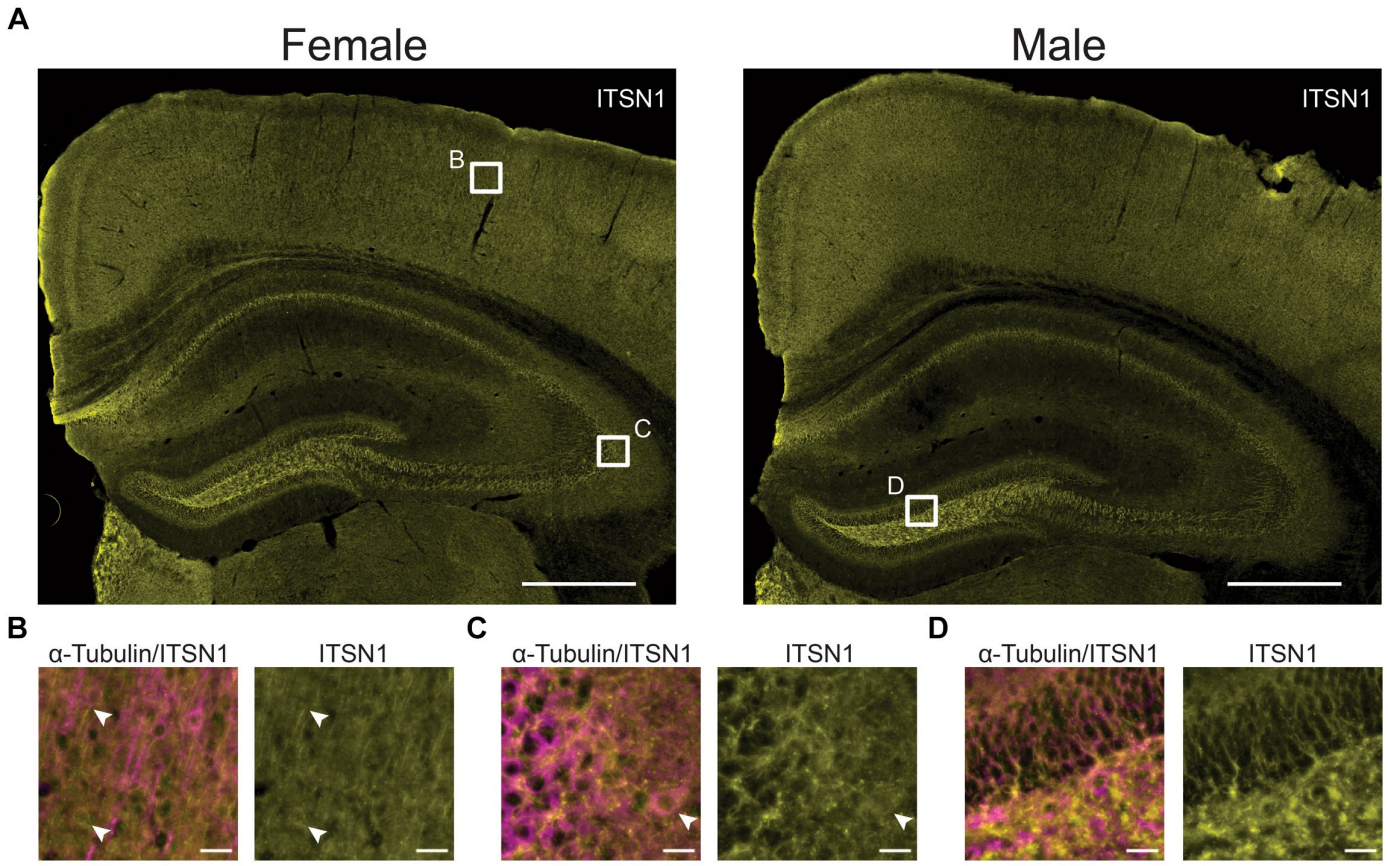
ITSN1 expression visualized in 9-month WT mice. **(A)** Representative wide field images of 9 month old male and female WT mouse brains stained for ITSN1 (yellow). White boxes in **(A)** indicate areas magnified in B, C, and D in the CTX **(B)**, HP CA3 **(C)**, and dentate gyrus **(D)**. Each region is shown with both neuronal α-Tubulin (magenta) and ITSN1 (yellow). Scale bars are 500 μm in **(A)** and 20 μm in **(B–D)**.

To evaluate whether ITSN1 localization changes between 5xFAD and WT mice at each timepoint, we performed immunofluorescence to detect ITSN1 and α-Tubulin, a neuronal marker which marks the axon and cell body (Nirschl et al., 2016; Guedes-Dias and Holzbaur, 2019). We did not observe any obvious differences in ITSN1 staining patterns between age and genotypes (Supplementary Figure S4). Generally, ITSN1 showed widespread staining across the tissue with some bright puncta in HP CA subfields and dentate gyrus (Figure 2A), but this was not always concentrated around the α-Tubulin neurons. In the CTX, ITSN1 was sometimes seen to be more concentrated in cell bodies not positive for α-Tubulin (Figure 2B, bottom white arrow) and in long processes adjacent to α-Tubulin positive cell bodies (Figure 2B, top white arrow). However, some cells that were α-Tubulin positive also contain concentrated ITSN1 near the nucleus (Figure 2C, white arrows).

### 3.6 ITSN1-L is decreased in the aged female 5xFAD CTX and HP

**Figure 3:**
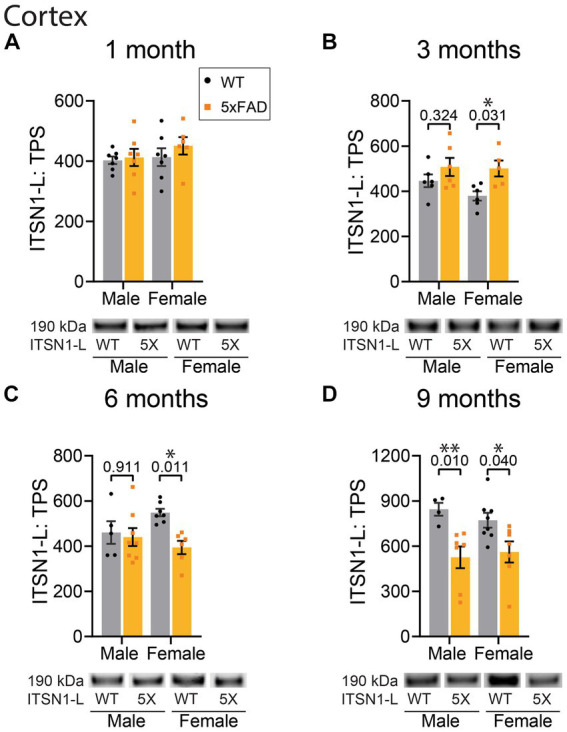
ITSN1 expression is increased in female 5xFAD CTX at 3 months but decreased at 6 and 9 months of age. Immunoblot comparison of ITSN1-L between males and females in 5xFAD and WT littermate controls in the CTX at 1, 3, 6, and 9 months of age. For each analysis, all graphs **(A–D)** show protein intensity values at 1, 3, 6, and 9 months normalized to total protein stain (TPS) with representative immunoblot bands underneath. Data are represented as mean ± SEM. Statistical significance was determined by two-way ANOVAs followed by Šídák multiple comparisons *post-hoc* tests for analyses with a significant main effect. *p < 0.05; **p < 0.01.

**Figure 4:**
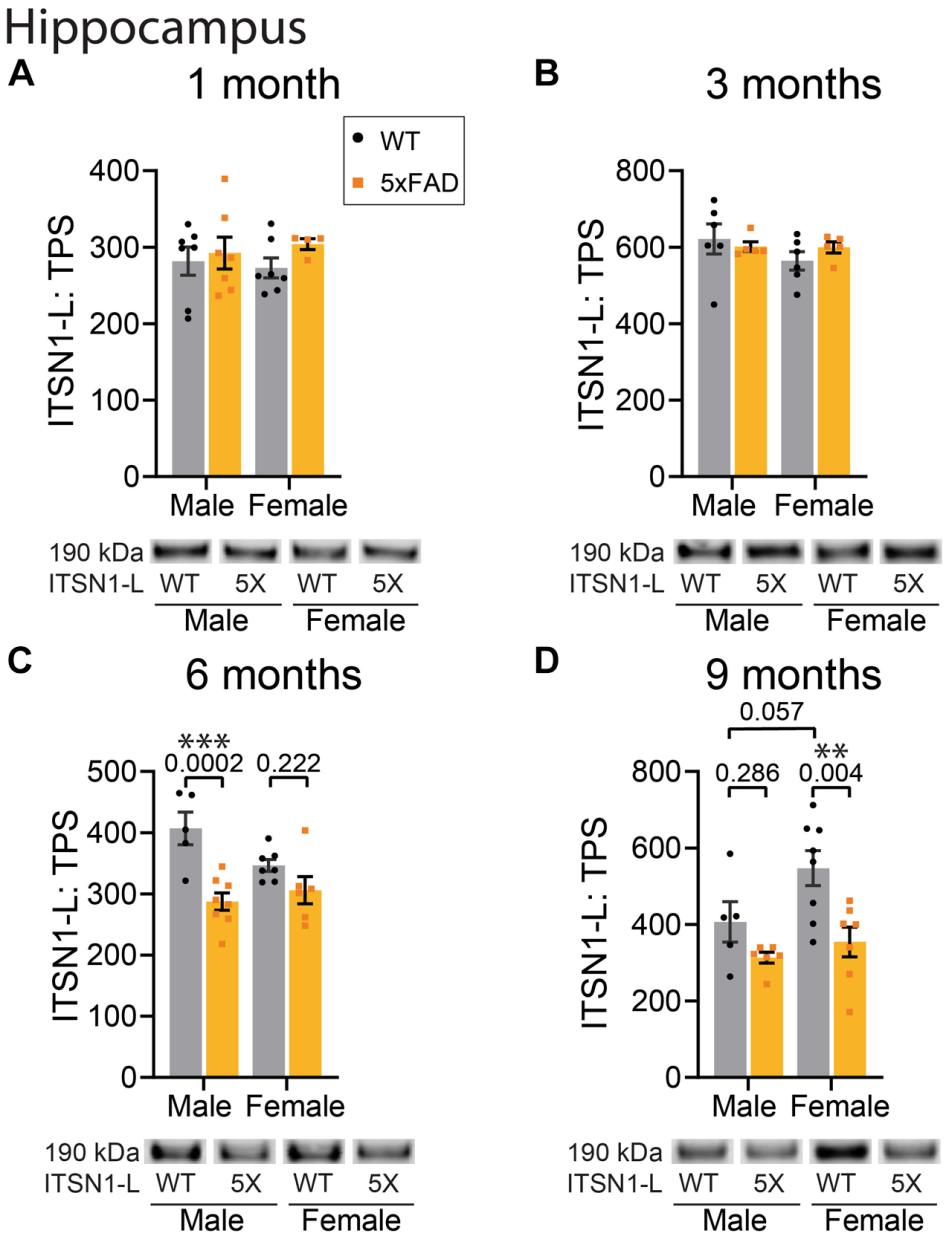
ITSN1-L is decreased in 5xFAD HP at 6 and 9 months of age. Immunoblot comparison of ITSN1-L between males and females in 5xFAD and WT littermate controls in the HP at 1, 3, 6, and 9 months of age. For each analysis, all graphs **(A–D)** show protein intensity values at 1, 3, 6, and 9 months normalized to total protein stain (TPS) with representative immunoblot bands underneath. Data are represented as mean ± SEM. Statistical significance was determined by two-way ANOVAs followed by Šídák multiple comparisons post-hoc tests for analyses with a significant main effect. **p < 0.01; ***p < 0.001.

To examine if the changes in ITSN1 we observed in human AD brains also occur in an AD mouse model with amyloid pathology, we performed immunoblots on the CTX and HP of 5xFAD and WT littermate controls. As ITSN1 is involved in neurodevelopment (Jakob et al., 2017) and AD pathology does not appear until adulthood, we examined ITSN1 levels between 5xFAD and WT at 1-, 3-, 6-, and 9-months in both males and females (Figures 3, 4). In mice, ITSN1-S was much less abundant than ITSN1-L and did not have high enough signal to noise ratio to measure by immunoblot.

In the CTX, there was no effect of genotype on ITSN1-L levels at 1 month of age (Figure 3A). However, at 3 months we found a significant effect of genotype on ITSN1-L (*p* = 0.0095) (Figure 3B). Subsequent post-hoc tests revealed a significant increase of ITSN1-L in 5xFAD females compared to CTL females (*p* = 0.031). At 6 months we again found a significant genotype effect on ITSN1-L (*p* = 0.023) with post-hoc tests now showing a significant decrease of ITSN1-L in 5xFAD females as compared to CTLs (*p* = 0.011) (Figure 3C). At 9 months, the significant genotype effect was sustained (*p* = 0.0006) and post-hoc tests showed that ITSN1-L significantly decreases in both male (*p* = 0.001) and female (*p* = 0.040) 5xFAD mice compared to sex matched CTLs (Figure 3D).

In the HP, there was no significant effect of genotype at 1 and 3 months (Figures 4A,B). However, at 6 months we observed a significant main effect of genotype (*p* = 0.0002) as well as a significant interaction between genotype and sex (*p* = 0.038) (Figure 4C). Post-hoc tests revealed a significant decrease of ITSN1 levels in 5xFAD males as compared to WT males (*p* = 0.0002). Interestingly, there was also a trending decrease in female WT mice compared to male WTs (*p* = 0.061) indicating a potential baseline difference between sexes. At 9 months, we found significant genotype (*p* = 0.0024) and sex (*p* = 0.041) effects on ITSN1-L levels (Figure 4D). Post-hoc tests showed that ITSN1-L is decreased in 5xFAD females (*p* = 0.004) and has a trending increase in female WT versus, male WT ITSN1-L levels (*p* = 0.057) (Figure 4D). Full statistical results for ITSN1 in the CTX and HP are reported in Supplementary Table S4.

**Figure 5:**
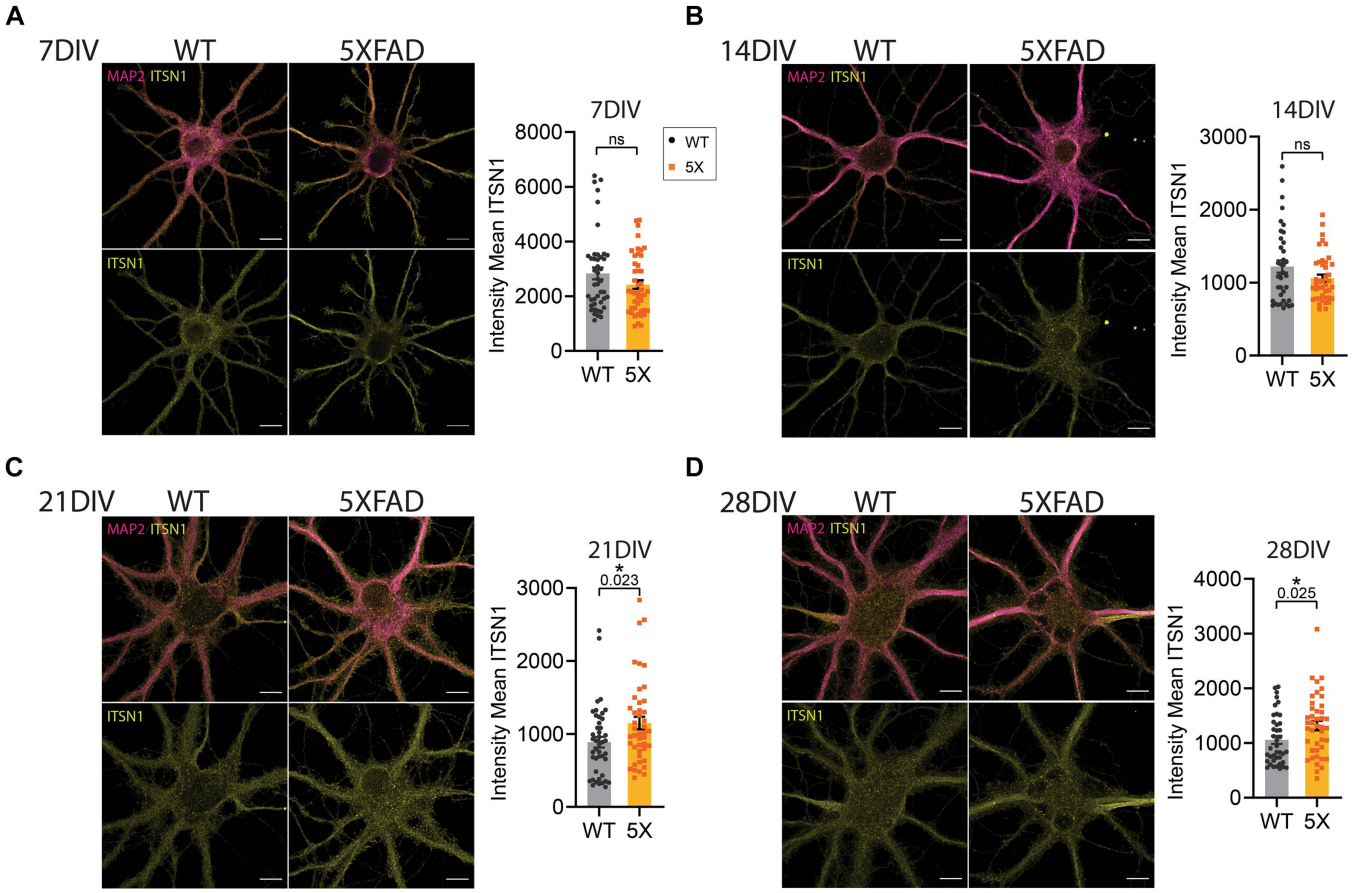
ITSN1 is increased in aged 5xFAD HP neurons. Confocal microscopy images of 5xFAD and WT primary HP neurons at 7 **(A)**, 14 **(B)**, 21 **(C)** and 28 **(D)** days *in vitro* (DIV). Both WT and 5xFAD neurons were stained with ITSN1-L (yellow) and microtubule associated protein 2 (MAP2, violet). Neurons were individually segmented by MAP2 using Zeiss Zen Intellesis trainable artificial intelligence segmentation. For each set of images, graphs show the intensity mean values of ITSN1 in the MAP2 segmented area with each data point corresponding to one neuron. Experiments were carried out independently 2–3 times per group and imaged across three coverslips per repetition for a total *n* = 39–45 cells per group. Data are represented as mean ± SEM. Statistical significance was determined by unpaired, two-tailed *t*-tests. *p < 0.05, all scale bars 10 μm.

### 3.7 Increased ITSN1 in 5xFAD aged HP neurons

To further evaluate the impact of aging on ITSN1 in 5xFAD neurons we grew primary HP neurons isolated from 5xFAD and WT E15 embryos in sandwich culture with glia (Kaech and Banker, 2006). We performed dual immunofluorescence of ITSN1 and MAP2 in 5xFAD and WT neurons at 7, 14, 21, and 28 DIV and imaged individual cells at each time-point. Analysis of staining in neurons has historically been challenging as traditional segmentation methods cannot automatically detect the intricate processes of a neuron. To mitigate this, we used trainable AI segmentation of MAP2 on a subset of images across time-points to identify the neuron and processes, which was then applied to all images to create an object that encompasses the neuronal cell body as well as dendrites. Mean ITSN1 signal was measured within the MAP2 segmentation and compared between 5xFAD and WT neurons. At 7 and 14 DIV, there were no significant differences in ITSN1 (Figures 5A,B). At 21 DIV, we observed a significant increase of ITSN1 in 5xFAD cells (*p* = 0.023) and again at 28 DIV (*p* = 0.025) (Figures 5C,D).

**Figure 6:**
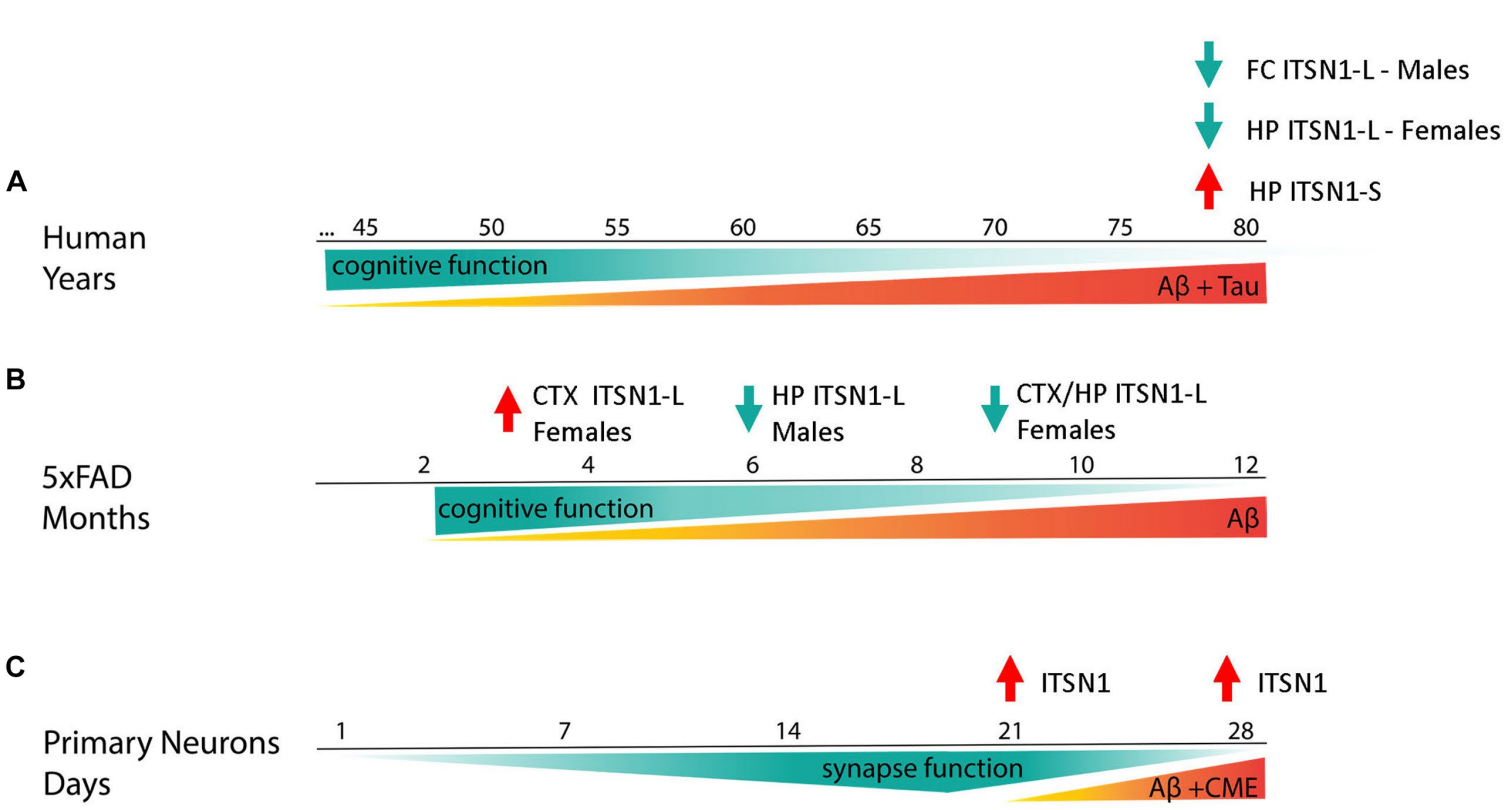
Summary figure. ITSN1-L and -S change in an isoform and brain region specific manner in post-mortem AD human brains **(A)**. In the HP, ITSN1-S is increased in both AD males and females whereas ITSN1-L is decreased only in AD females. In the FC, ITSN1-L is only decreased in AD males. These changes in ITSN1-L are somewhat recapitulated in the 5xFAD model **(B)**. Interestingly, our “early stage” models of AD, 5xFAD at 3 months and 5xFAD primary neurons **(C)**, capture disease states earlier than possible with human post-mortem tissue and show an increase in ITSN1-L illustrating a potential transient mechanism in which ITSN1-L is increased during early-stage disease and decreased during late-stage disease.

## 4 Discussion

The goal of this study was to use candidate AD miRNA biomarkers to discover and evaluate potential pathways contributing to AD mechanisms. We found that CME may be regulated by the candidate AD miRNAs and examined several CME proteins based on their existing association with AD, their potential to be regulated by the AD CSF candidate biomarkers, and how essential they are in the pathway. Our study is the first to perform a comprehensive evaluation of major CME hub proteins in the same cohort of samples and multiple brain regions. By immunoblot, protein levels of the scaffolding protein ITSN1 showed isoform and brain region specific differences between post-mortem human AD and CTL brains, with decreases in the FC of AD males and HP of AD females (Figure 1). However, there were no appreciable differences between AD and CTL in the other CME proteins assessed herein (Supplementary Figures S1, S2). Additionally, we measured ITSN1 in the 5xFAD mouse brain throughout aging because a mouse model that recapitulates human ITSN1 expression levels could be used to mechanistically examine CME and other ITSN1 functions in the context of AD. By immunoblot we found that in the 5xFAD mice, ITSN1-L in the CTX is increased in females at 3 months but decreased at 6 and 9 months of age, while in the HP there was decrease in ITSN1-L at 9 months in both female and male mice. The reduction of ITSN1-L in aged 5xFAD mice is similar to our results from end stage AD in human post-mortem brain and implicates a role for Aβ accumulation and pathology in ITSN1-L dysregulation. Interestingly, the 5xFAD primary neurons showed increased ITSN1-L compared to WT upon neuron maturation at 21 and 28 DIV, which recapitulates the transient increase in the CTX of 3 month old 5xFAD females when Aβ plaques are beginning to develop (Oakley et al., 2006). Together these studies suggest that amyloidosis triggers changes in ITSN1-L expression in the brain, which may impact CME and/or synaptic transmission.

Thus far, few studies have evaluated ITSN1 in the context of AD in humans. At the mRNA level, ITSN1-S was upregulated in human post-mortem AD FC, but ITSN1-L showed no expression changes (Wilmot et al., 2008). Another transcriptomic study found ITSN1 mRNA upregulated in AD temporal CTX with increasing neurofibrillary tangle pathology (Dunckley et al., 2006) though the isoform was not specified. The only study that previously evaluated ITSN1 protein levels in AD brains found no difference in frontal or temporal CTX compared to CTL in either isoform (Malakooti, 2019). In contrast, our study revealed that in AD, ITSN1 isoform expression is altered in a brain region-and sex-dependent manner. In the FC, ITSN1-S expression was too low for analysis. However, in the HP both AD females and males had similar changes in ITSN1-S compared to CTL, with a significant increase in AD females and trend of increased expression in AD males. For ITSN1-L, in the FC there was a decrease in AD males, but no change in AD females. Conversely, in the HP ITSN1-L was increased in AD females, but not different between AD and CTL males. However, it is important to note that CTL male brains had very low levels of ITSN1-L in the HP. Thus, more sensitive detection methods are needed to confirm our finding that there is no significant difference in ITSN1-L levels between AD and CTL males. Additionally, we observed that the HP female CTLs have a higher baseline of ITSN1-L levels than CTL males, which suggests that protein expression levels in normal aging females versus males is sex-dependent. This is interesting as females have higher instances of AD (Rajan et al., 2021; 2023) and the decrease in ITSN1-L we observe in AD female HP could be connected to their increased vulnerability to neurodegeneration. Our data may differ from the previous protein study due to differences in male to female ratios of the AD and CTL groups, with the previous study’s AD group being primarily male, and combining sexes within groups (Malakooti, 2019), whereas we evaluated sexes independently. Together, these data show that both sex and brain region should be taken into consideration when assessing protein changes as a result of AD.

To begin to elucidate what pathological mechanism(s) (e.g., Aβ vs. Tau) impact ITSN1 changes in AD, we evaluated ITSN1 levels in the 5xFAD mouse model over the lifespan. 5xFAD mice contain three AD familial mutations in humanized *APP* and two in *PSEN1* (Goate et al., 1991; Mullan et al., 1992; Eckman et al., 1997; Citron et al., 1998; Berezovska et al., 2005), and exhibit Aβ plaques, neurodegeneration, and cognitive decline as they age (Oakley et al., 2006; Jawhar et al., 2012; Forner et al., 2021; Oblak et al., 2021). Differences in protein expression between 5xFAD and WT mice can be seen as early as postnatal day 1, and pathway analysis of these genes revealed changes in CME (Mazi et al., 2018). We found that ITSN1-S was not abundant enough to measure by immunoblot, but we did observe changes in ITSN1-L at different time-points across the study timeline.

In the CTX, we saw an increase of ITSN1-L in female 5xFAD mice at 3 months. This is interesting as female 5xFAD mice exhibit plaque pathology earlier than males (Forner et al., 2021) which could suggest that an increase in ITSN1-L is associated with the increase of Aβ burden in early stages of pathology development in female 5xFAD mice. However, at later timepoints (6, 9 months) this increase in ITSN1-L is not sustained and transitions to a decrease in ITSN1-L in both the CTX and HP of female mice by 9 months. 5xFAD mice begin extracellular deposition of plaques between 2 and 4 months and plaques first appear in the subiculum and cortical layer V (Oakley et al., 2006). It is possible that early in the disease, ITSN1-L changes in 5xFAD, like the cortical increase we saw at 3-months, have a different effect on pathology progression than changes at 6 months, where we see ITSN1-L decrease. An increase at 3 months could correspond with the increase in Aβ plaques in the CTX (Oakley et al., 2006) and because changes in ITSN1 levels are likely to reduce CME (Sengar, 1999; Simpson et al., 1999; Yu et al., 2008; Thomas et al., 2009), this could suggest a transient mechanism by which ITSN1-L is compensatory early in disease to combat the effects of increased Aβ burden by decreasing APP uptake.

In pursuit of an *in vitro* model to examine ITSN1 changes mechanistically, we compared ITSN1 levels via immunofluorescence in 5xFAD and WT primary HP neurons. We found that ITSN1 was significantly increased in 5xFAD neurons at 21 and 28 DIV in these mixed sex cultures. As previously shown in WT primary cortical neurons, CME and production of APP is increased in 28 DIV ‘aged’ neurons compared to younger neurons (Burrinha et al., 2021). The same study also showed that inhibition of CME limits Aβ production which reverts *in vitro* age-related synaptic loss (Burrinha et al., 2021). Thus, the increase we saw in ITSN1 at 21 and 28 DIV 5xFAD primary HP neurons, which corresponds to increased CME and APP production in WT neurons, and tracks with accumulation of Aβ oligomers in 5xFAD primary neurons seen after 14 days (Shim et al., 2021), could also function as a compensatory mechanism to reduce CME and Aβ and prevent subsequent synaptic loss. This further supports the need for future mechanistic studies to examine the hypothesis that the early ITSN1-L increase in 5xFAD females is a protective response to Aβ. Also, the use of a single sex primary culture would be interesting to evaluate potential mechanistic differences due to ITSN1 changes in each sex individually. Studies using 5xFAD primary neurons are also needed to provide clarification on the specific cellular mechanisms, CME or otherwise, that are affected by ITSN1 expression changed in AD.

It is interesting that in the HP of 9-month-old 5xFAD mice we see the same pattern seen in human post-mortem AD HP; a trending increase in baseline level of ITSN1-L in WT female mice compared to WT males and a decrease of ITSN1-L in HP of 5xFAD females compared to WT females. These data suggest that ITSN1-L function may be associated with pathology progression in the 5xFAD model and that aged mice recapitulate ITSN1-L changes seen in end-stage disease in humans. Thus, further characterization of these ITSN1-L changes in the 5xFAD HP may be relevant to discerning the mechanism underlying human AD.

As age is the highest risk factor for AD (Guerreiro and Bras, 2015), CME proteins increase with age (Alsaqati et al., 2018), and ITSN1 itself is increased with age in as shown in a Down Syndrome (DS) mouse model (Ahmed et al., 2017), there is a high likelihood that age-related changes may play an important role in ITSN1 function and dysfunction in AD. Increased ITSN1-L with aging could even be protective and loss of that protection from decreased ITSN1-L in aged 5xFAD mice may contribute to further disease progression. While we cannot compare ITSN1-L levels between timepoints in our mouse immunoblots or primary neurons due to batch effects of processing each timepoint individually, future studies should examine the relationship between age, genotype, and sex effects on ITSN1-L in 5xFAD mice.

Our findings of increased ITSN1-S in human AD postmortem brain do not support the idea that a general decrease in ITSN1 contributes to AD. The difference in ITSN1-S and -L levels in AD point to their having distinct functions in each brain region. ITSN1-S is only involved in CME and has identical functional domains as ITSN1-L that interact with CME proteins. However, ITSN1-L has several additional neuronal specific functions due to the three additional binding domains at the C-terminus (Tsyba et al., 2008, 2011). These functions include dendritic spine development (Irie and Yamaguchi, 2002; Kuboyama et al., 2015), fast neurotransmission (Sakaba et al., 2013; Jäpel et al., 2020), mitogenic signaling (Adams et al., 2000; O’Bryan et al., 2001), and RAS/MAPK regulation (Tong et al., 2000). So, while the increase of ITSN1-S most likely affects only cellular functions which use CME machinery, the decrease of ITSN1-L may not involve CME at all. The differences between isoform function, specifically which CME processes ITSN1-S is involved in versus those that use ITSN1-L, have not been studied. Our data suggest that future studies should make efforts to distinguish how changes in each isoform affect specific cellular functions. As a first step, discovering which cell types are driving ITSN1-S versus ITSN-L changes may help to narrow down each isoforms specific function in the affected cell type.

Of the other CME proteins we evaluated (Supplementary Figures S1, S2), DNM2 (Aidaralieva et al., 2008; Kamagata et al., 2009), AP2A1 (Yao et al., 2000), CLTA (Nakamura et al., 1994), and PICALM (Ando et al., 2013, 2016; Alsaqati et al., 2023) have been shown to exhibit various protein, RNA, or localization changes associated with AD. While we observed no changes by immunoblot in any of these proteins, our study differed from many of these in several ways. Of particular interest, our analysis of PICALM levels does not recapitulate previous studies, which show PICALM isoform 4 as both increased (Ando et al., 2016) or decreased (Ando et al., 2013; Alsaqati et al., 2023) in AD depending on if the soluble or insoluble protein fraction was extracted. As our sample preparation protocol captures both soluble and insoluble protein fractions, we may not capture the difference in PICALM levels seen previously in different fractions. However, the prior studies primarily used male AD brains due to the challenges of sample access. Our inclusion of both males and females allowed us to observe a trending interaction between sex and disease on PICALM isoform 4 levels (*p* = 0.051) in the HP. This points to a potential difference in how PICALM isoform 4 levels may change differently in AD between males and females.

In conclusion, the pathophysiology of AD is extremely complex, affecting many cell types, brain regions, and higher order brain functions. This study illustrates how target prediction of candidate miRNA AD biomarkers can help identify AD related cellular pathways. Here, we began to evaluate one of these pathways, CME, for specific changes in AD using human post-mortem brains, the 5xFAD mouse model, and primary neuronal culture. Of the CME proteins we assayed, ITSN1 was revealed as the only protein significantly changed in AD postmortem brains. Isoform and brain region specific changes of ITSN1, in human AD brains which are in part recapitulated in 5xFAD mice, suggest distinct functions of ITSN1-L and -S and the possibility of different ITSN1-L functions in early versus late disease stages. As summarized in Figure 6, timepoints of earlier disease states, modeled by 3-month female 5xFAD mice and primary 5xFAD neurons, show an increase in ITSN1-L. While time points during later stages of disease, modeled by post-mortem human AD brains and aged 5xFAD mice, show a decrease in ITSN1-L. Our results develop a compelling reason to further investigate specific ITSN1-L and -S functions in AD over disease progression, while also pointing out that future studies must be carefully designed with specific models that can capture brain region, isoform, and sex differences at different time points of the disease.

## Data availability statement

The original contributions presented in the study are included in the article/Supplementary material, further inquiries can be directed to the corresponding author.

## Ethics statement

The animal study was approved by the OHSU Institutional Animal Care and Use Committee. The study was conducted in accordance with the local legislation and institutional requirements.

## Author contributions

SJ: Writing – review & editing, Conceptualization, Formal analysis, Funding acquisition, Investigation, Writing – original draft, Data curation, Methodology. US: Conceptualization, Formal analysis, Writing – review & editing, Methodology, Supervision. TM: Methodology, Writing – review & editing, Investigation. RW: Methodology, Resources, Writing – review & editing, Data curation. JS: Conceptualization, Supervision, Validation, Writing – review & editing, Funding acquisition.

## References

[ref1] Adams A. Thorn J. M. Yamabhai M. Kay B. K. Obryan J. P. (2000). Intersectin, an adaptor protein involved in clathrin-mediated endocytosis, activates mitogenic signaling pathways. J. Biol. Chem. 275, 27414–27420. doi: 10.1016/S0021-9258(19)61526-7, PMID: 10851244

[ref2] Ahmed M. M. Block A. Tong S. Davisson M. T. Gardiner K. J. (2017). Age exacerbates abnormal protein expression in a mouse model of down syndrome. Neurobiol. Aging 57, 120–132. doi: 10.1016/j.neurobiolaging.2017.05.002, PMID: 28641136

[ref3] Aidaralieva N. J. Kamino K. Kimura R. Yamamoto M. Morihara T. Kazui H.. (2008). Dynamin 2 gene is a novel susceptibility gene for late-onset Alzheimer disease in non-APOE-ε4 carriers. J. Hum. Genet. 53, 296–302. doi: 10.1007/s10038-008-0251-9, PMID: 18236001

[ref4] Alsaqati M. Thomas R. S. Kidd E. J. (2018). Proteins involved in endocytosis are upregulated by ageing in the Normal human brain: implications for the development of Alzheimer’s disease. J. Gerontol. Ser. A 73, 289–298. doi: 10.1093/gerona/glx135, PMID: 28655199

[ref5] Alsaqati M. Thomas R. S. Kidd E. J. (2023). Upregulation of endocytic protein expression in the Alzheimer's disease male human brain. Aging Brain 4:100084. doi: 10.1016/j.nbas.2023.100084, PMID: 37449017 PMC10336166

[ref6] Alzheimer’s Association (2023). 2023 Alzheimer’s disease facts and figures. Alzheimers Dement. 19, 1598–1695. doi: 10.1002/alz.13016 36918389

[ref7] Ando K. Brion J. P. Stygelbout V. Suain V. Authelet M. Dedecker R.. (2013). Clathrin adaptor CALM/PICALM is associated with neurofibrillary tangles and is cleaved in Alzheimer's brains. Acta Neuropathol. 125, 861–878. doi: 10.1007/s00401-013-1111-z, PMID: 23589030

[ref8] Ando K. Tomimura K. Sazdovitch V. Suain V. Yilmaz Z. Authelet M.. (2016). Level of PICALM, a key component of clathrin-mediated endocytosis, is correlated with levels of phosphotau and autophagy-related proteins and is associated with tau inclusions in AD, PSP and pick disease. Neurobiol. Dis. 94, 32–43. doi: 10.1016/j.nbd.2016.05.017, PMID: 27260836

[ref9] Azarnia T. López H. Maritzen T. (2019). Endocytic adaptor proteins in health and disease: lessons from model organisms and human mutations. Cells 8:1345. doi: 10.3390/cells8111345, PMID: 31671891 PMC6912373

[ref10] Berezovska O. Lleo A. Herl L. D. Frosch M. P. Stern E. A. Bacskai B. J.. (2005). Familial Alzheimer's disease presenilin 1 mutations cause alterations in the conformation of presenilin and interactions with amyloid precursor protein. J. Neurosci. 25, 3009–3017. doi: 10.1523/JNEUROSCI.0364-05.2005, PMID: 15772361 PMC6725136

[ref11] Braak H. Braak E. (1991). Neuropathological stageing of Alzheimer-related changes. Acta Neuropathol. 82, 239–259. doi: 10.1007/BF00308809, PMID: 1759558

[ref12] Burrinha T. Martinsson I. Gomes R. Terrasso A. P. Gouras G. K. Almeida C. G. (2021). Upregulation of APP endocytosis by neuronal aging drives amyloid-dependent synapse loss. J. Cell Sci. 134:jcs255752. doi: 10.1242/jcs.255752, PMID: 33910234

[ref13] Cao Y. Xiao Y. Ravid R. Guan Z. Z. (2010). Changed clathrin regulatory proteins in the brains of Alzheimer's disease patients and animal models. J. Alzheimers Dis. 22, 329–342. doi: 10.3233/JAD-2010-100162, PMID: 20847448

[ref14] Cataldo A. M. Barnett J. L. Pieroni C. Nixon R. A. (1997). Increased neuronal endocytosis and protease delivery to early endosomes in sporadic Alzheimer’s disease: neuropathologic evidence for a mechanism of increased β-Amyloidogenesis. J. Neurosci. 17, 6142–6151. doi: 10.1523/JNEUROSCI.17-16-06142.1997, PMID: 9236226 PMC6568334

[ref15] Cataldo A. M. Peterhoff C. M. Troncoso J. C. Gomez-Isla T. Hyman B. T. Nixon R. A. (2000). Endocytic pathway abnormalities precede amyloid β deposition in sporadic Alzheimer’s disease and down syndrome. Am. J. Pathol. 157, 277–286. doi: 10.1016/S0002-9440(10)64538-5, PMID: 10880397 PMC1850219

[ref16] Cirrito J. R. Kang J.-E. Lee J. Stewart F. R. Verges D. K. Silverio L. M.. (2008). Endocytosis is required for synaptic activity-dependent release of amyloid-β *in vivo*. Neuron 58, 42–51. doi: 10.1016/j.neuron.2008.02.003, PMID: 18400162 PMC2390913

[ref17] Citron M. Eckman C. B. Diehl T. S. Corcoran C. Ostaszewski B. L. Xia W.. (1998). Additive effects of PS1 and APP mutations on secretion of the 42-residue amyloid β-protein. Neurobiol. Dis. 5, 107–116. doi: 10.1006/nbdi.1998.0183, PMID: 9746908

[ref18] Colacurcio D. J. Pensalfini A. Jiang Y. Nixon R. A. (2018). Dysfunction of autophagy and endosomal-lysosomal pathways: roles in pathogenesis of down syndrome and Alzheimer's disease. Free Radic. Biol. Med. 114, 40–51. doi: 10.1016/j.freeradbiomed.2017.10.001, PMID: 28988799 PMC5748263

[ref19] Deture M. A. Dickson D. W. (2019). The neuropathological diagnosis of Alzheimer’s disease. Mol. Neurodegener. 14:32. doi: 10.1186/s13024-019-0333-5, PMID: 31375134 PMC6679484

[ref20] Doherty G. J. Mcmahon H. T. (2009). Mechanisms of endocytosis. Annu. Rev. Biochem. 78, 857–902. doi: 10.1146/annurev.biochem.78.081307.110540, PMID: 19317650

[ref21] Du S. S. Sun X. Cen J. Shi J. X. An M. X. Zhao W. D. (2021). Dynamin-2 mediates clathrin-dependent endocytosis for amyloid-beta internalization in brain microvascular endothelial cells. Microvasc. Res. 138:104219. doi: 10.1016/j.mvr.2021.104219, PMID: 34214572

[ref22] Dunckley T. Beach T. G. Ramsey K. E. Grover A. Mastroeni D. Walker D. G.. (2006). Gene expression correlates of neurofibrillary tangles in Alzheimer's disease. Neurobiol. Aging 27, 1359–1371. doi: 10.1016/j.neurobiolaging.2005.08.013 16242812 PMC2259291

[ref23] Eckman C. B. Mehta N. D. Crook R. Perez-Tur J. Prihar G. Pfeiffer E.. (1997). A new pathogenic mutation in the APP gene (I716V) increases the relative proportion of a beta 42 (43). Hum. Mol. Genet. 6, 2087–2089. doi: 10.1093/hmg/6.12.2087, PMID: 9328472

[ref24] Filipowicz W. Bhattacharyya S. N. Sonenberg N. (2008). Mechanisms of post-transcriptional regulation by microRNAs: are the answers in sight? Nat. Rev. Genet. 9, 102–114. doi: 10.1038/nrg2290, PMID: 18197166

[ref25] Forner S. Kawauchi S. Balderrama-Gutierrez G. Kramár E. A. Matheos D. P. Phan J.. (2021). Systematic phenotyping and characterization of the 5xFAD mouse model of Alzheimer’s disease. Scientific Data 8:270. doi: 10.1038/s41597-021-01054-y, PMID: 34654824 PMC8519958

[ref26] Gebert L. F. R. Macrae I. J. (2019). Regulation of microRNA function in animals. Nat. Rev. Mol. Cell Biol. 20, 21–37. doi: 10.1038/s41580-018-0045-7, PMID: 30108335 PMC6546304

[ref27] Goate A. Chartier-Harlin M. C. Mullan M. Brown J. Crawford F. Fidani L.. (1991). Segregation of a missense mutation in the amyloid precursor protein gene with familial Alzheimer's disease. Nature 349, 704–706. doi: 10.1038/349704a0, PMID: 1671712

[ref28] Gubar O. Morderer D. Tsyba L. Croisé P. Houy S. Ory S.. (2013). Intersectin: the crossroad between vesicle exocytosis and endocytosis. Front. Endocrinol. 4:109. doi: 10.3389/fendo.2013.00109, PMID: 23986746 PMC3753573

[ref29] Guedes-Dias P. Holzbaur E. L. F. (2019). Axonal transport: driving synaptic function. Science 366:eaaw9997. doi: 10.1126/science.aaw9997, PMID: 31601744 PMC6996143

[ref30] Guerreiro R. Bras J. (2015). The age factor in Alzheimer's disease. Genome Med. 7:106. doi: 10.1186/s13073-015-0232-5 26482651 PMC4617238

[ref31] Guntupalli S. Jang S. E. Zhu T. Huganir R. L. Widagdo J. Anggono V. (2017). GluA1 subunit ubiquitination mediates amyloid-β-induced loss of surface α-amino-3-hydroxy-5-methyl-4-isoxazolepropionic acid (AMPA) receptors. J. Biol. Chem. 292, 8186–8194. doi: 10.1074/jbc.M116.774554, PMID: 28377502 PMC5437227

[ref32] Herrero-Garcia E. O'bryan J. P. (2017). Intersectin scaffold proteins and their role in cell signaling and endocytosis. Biochim. Biophys. Acta 1864, 23–30. doi: 10.1016/j.bbamcr.2016.10.005 PMC5148706 27746143

[ref33] Hussain N. K. Yamabhai M. Ramjaun A. R. Guy A. M. Baranes D. Obryan J. P.. (1999). Splice variants of Intersectin are components of the endocytic machinery in neurons and nonneuronal cells. J. Biol. Chem. 274, 15671–15677. doi: 10.1074/jbc.274.22.15671, PMID: 10336464

[ref34] Irie F. Yamaguchi Y. (2002). EphB receptors regulate dendritic spine development via intersectin, Cdc42 and N-WASP. Nat. Neurosci. 5, 1117–1118. doi: 10.1038/nn964, PMID: 12389031

[ref35] Ishizuka Y. Bramham C. R. (2020). A simple DMSO-based method for cryopreservation of primary hippocampal and cortical neurons. J. Neurosci. Methods 333:108578. doi: 10.1016/j.jneumeth.2019.108578, PMID: 31899209

[ref36] Jacobs J. Kahana M. J. (2010). Direct brain recordings fuel advances in cognitive electrophysiology. Trends Cogn. Sci. 14, 162–171. doi: 10.1016/j.tics.2010.01.005, PMID: 20189441 PMC2847661

[ref37] Jakob B. Kochlamazashvili G. Jäpel M. Gauhar A. Bock H. H. Maritzen T.. (2017). Intersectin 1 is a component of the Reelin pathway to regulate neuronal migration and synaptic plasticity in the hippocampus. Proc. Natl. Acad. Sci. 114, 5533–5538. doi: 10.1073/pnas.1704447114, PMID: 28484035 PMC5448185

[ref38] Jäpel M. Gerth F. Sakaba T. Bacetic J. Yao L. Koo S.-J.. (2020). Intersectin-mediated clearance of SNARE complexes is required for fast neurotransmission. Cell Rep. 30, 409–420.e406. doi: 10.1016/j.celrep.2019.12.035, PMID: 31940485

[ref39] Jawhar S. Trawicka A. Jenneckens C. Bayer T. A. Wirths O. (2012). Motor deficits, neuron loss, and reduced anxiety coinciding with axonal degeneration and intraneuronal Abeta aggregation in the 5XFAD mouse model of Alzheimer's disease. Neurobiol. Aging 33, e129–e140. doi: 10.1016/j.neurobiolaging.2010.05.027 20619937

[ref40] Jaye S. Sandau U. S. Saugstad J. A. (2024). Clathrin mediated endocytosis in Alzheimer’s disease: cell type specific involvement in amyloid beta pathology. Front Aging Neurosci 16:1378576. doi: 10.3389/fnagi.2024.1378576 38694257 PMC11061891

[ref41] Kaech S. Banker G. (2006). Culturing hippocampal neurons. Nat. Protoc. 1, 2406–2415. doi: 10.1038/nprot.2006.356, PMID: 17406484

[ref42] Kaksonen M. Roux A. (2018). Mechanisms of clathrin-mediated endocytosis. Nat. Rev. Mol. Cell Biol. 19, 313–326. doi: 10.1038/nrm.2017.132, PMID: 29410531

[ref43] Kamagata E. Kudo T. Kimura R. Tanimukai H. Morihara T. Sadik M. G.. (2009). Decrease of dynamin 2 levels in late-onset Alzheimer's disease alters Abeta metabolism. Biochem. Biophys. Res. Commun. 379, 691–695. doi: 10.1016/j.bbrc.2008.12.147, PMID: 19126407

[ref44] Koo E. Squazzo S. (1994). Evidence that production and release of amyloid @protein involves the endocytic pathway. J. Biol. Chem. 269, 17386–17389. doi: 10.1016/S0021-9258(17)32449-3, PMID: 8021238

[ref45] Kuboyama T. Lee Y.-A. Nishiko H. Tohda C. (2015). Inhibition of clathrin-mediated endocytosis prevents amyloid β-induced axonal damage. Neurobiol. Aging 36, 1808–1819. doi: 10.1016/j.neurobiolaging.2015.02.005, PMID: 25772059

[ref46] Lacor P. N. Buniel M. C. Chang L. Fernandez S. J. Gong Y. Viola K. L.. (2004). Synaptic targeting by Alzheimer's-related amyloid beta oligomers. J. Neurosci. 24, 10191–10200. doi: 10.1523/JNEUROSCI.3432-04.2004, PMID: 15537891 PMC6730194

[ref47] Lusardi T. A. Phillips J. I. Wiedrick J. T. Harrington C. A. Lind B. Lapidus J. A.. (2016). MicroRNAs in human cerebrospinal fluid as biomarkers for Alzheimer’s disease. J. Alzheimers Dis. 55, 1223–1233. doi: 10.3233/JAD-160835, PMID: 27814298 PMC5587208

[ref48] Malakooti N. (2019). The down syndrome-associated protein, regulator of Calcineurin-1, is altered in Alzheimer's disease and dementia with Lewy bodies. J. Alzheimers Dis. Parkinsonism 9:462. doi: 10.4172/2161-0460.1000462 31263630 PMC6602587

[ref49] Malakooti N. Pritchard M. A. Chen F. Yu Y. Sgambelloni C. Adlard P. A.. (2020). The long isoform of Intersectin-1 has a role in learning and memory. Front. Behav. Neurosci. 14:24. doi: 10.3389/fnbeh.2020.00024, PMID: 32161523 PMC7052523

[ref50] Man H.-Y. Lin J. W. Ju W. H. Ahmadian G. Liu L. Becker L. E.. (2000). Regulation of AMPA receptor–mediated synaptic transmission by Clathrin-dependent receptor internalization. Neuron 25, 649–662. doi: 10.1016/S0896-6273(00)81067-3, PMID: 10774732

[ref51] Mazi A. R. Arzuman A. S. Gurel B. Sahin B. Tuzuner M. B. Ozansoy M.. (2018). Neonatal neurodegeneration in Alzheimer's disease transgenic mouse model. J. Alzheimers Dis. Rep. 2, 79–91. doi: 10.3233/ADR-170049 30480251 PMC6159732

[ref52] Mcmahon H. T. Boucrot E. (2011). Molecular mechanism and physiological functions of clathrin-mediated endocytosis. Nat. Rev. Mol. Cell Biol. 12, 517–533. doi: 10.1038/nrm3151, PMID: 21779028

[ref53] Milosevic I. (2018). Revisiting the role of Clathrin-mediated Endoytosis in synaptic vesicle recycling. Front. Cell. Neurosci. 12:27. doi: 10.3389/fncel.2018.00027, PMID: 29467622 PMC5807904

[ref54] Motulsky H. J. Brown R. E. (2006). Detecting outliers when fitting data with nonlinear regression – a new method based on robust nonlinear regression and the false discovery rate. BMC Bioinformatics 7:123. doi: 10.1186/1471-2105-7-123 16526949 PMC1472692

[ref55] Mullan M. Crawford F. Axelman K. Houlden H. Lilius L. Winblad B.. (1992). A pathogenic mutation for probable Alzheimer's disease in the APP gene at the N-terminus of beta-amyloid. Nat. Genet. 1, 345–347. doi: 10.1038/ng0892-345, PMID: 1302033

[ref56] Nakamura Y. Takeda M. Yoshimi K. Hattori H. Hariguchi S. Kitajima S.. (1994). Involvement of clathrin light chains in the pathology of Alzheimer's disease. Acta Neuropathol. 87, 23–31. doi: 10.1007/BF00386251, PMID: 8140893

[ref57] Narayan P. Sienski G. Bonner J. M. Lin Y.-T. Seo J. Baru V.. (2020). PICALM rescues endocytic defects caused by the Alzheimer’s disease risk factor APOE4. Cell Rep. 33:108224. doi: 10.1016/j.celrep.2020.108224, PMID: 33027662 PMC8190562

[ref58] Nelson J. W. Young J. M. Borkar R. N. Woltjer R. L. Quinn J. F. Silbert L. C.. (2014). Role of soluble epoxide hydrolase in age-related vascular cognitive decline. Prostaglandins Other Lipid Mediat 113–115, 30–37. doi: 10.1016/j.prostaglandins.2014.09.003, PMID: 25277097 PMC4254026

[ref59] Nirschl J. J. Magiera M. M. Lazarus J. E. Janke C. Holzbaur E. L. F. (2016). α-Tubulin Tyrosination and CLIP-170 phosphorylation regulate the initiation of dynein-driven transport in neurons. Cell Rep. 14, 2637–2652. doi: 10.1016/j.celrep.2016.02.046, PMID: 26972003 PMC4819336

[ref60] Nixon R. A. (2005). Endosome function and dysfunction in Alzheimer's disease and other neurodegenerative diseases. Neurobiol. Aging 26, 373–382. doi: 10.1016/j.neurobiolaging.2004.09.018, PMID: 15639316

[ref61] Nixon R. A. (2017). Amyloid precursor protein and endosomal-lysosomal dysfunction in Alzheimer's disease: inseparable partners in a multifactorial disease. FASEB J. 31, 2729–2743. doi: 10.1096/fj.201700359, PMID: 28663518 PMC6137496

[ref62] O’Bryan J. P. Mohney R. P. Oldham C. E. (2001). Mitogenesis and endocytosis: what's at the INTERSECTIoN? Oncogene 20, 6300–6308. doi: 10.1038/sj.onc.1204773, PMID: 11607832

[ref63] Oakley H. Cole S. L. Logan S. Maus E. Shao P. Craft J.. (2006). Intraneuronal β-amyloid aggregates, neurodegeneration, and neuron loss in transgenic mice with five familial Alzheimer's disease mutations: potential factors in amyloid plaque formation. J. Neurosci. 26, 10129–10140. doi: 10.1523/JNEUROSCI.1202-06.2006, PMID: 17021169 PMC6674618

[ref64] Oblak A. L. Lin P. B. Kotredes K. P. Pandey R. S. Garceau D. Williams H. M.. (2021). Comprehensive evaluation of the 5XFAD mouse model for preclinical testing applications: a MODEL-AD study. Front. Aging Neurosci. 13:713726. doi: 10.3389/fnagi.2021.713726, PMID: 34366832 PMC8346252

[ref65] Okamoto M. Schoch S. Südhof T. C. (1999). EHSH1/Intersectin, a protein that cintains EH and SH3 domais and binds ti dynamin and SNAP-25. J. Biol. Chem. 274, 18446–18454. doi: 10.1074/jbc.274.26.18446 10373452

[ref66] Oughtred R. Rust J. Chang C. Breitkreutz B. J. Stark C. Willems A.. (2021). The BioGRID database: a comprehensive biomedical resource of curated protein, genetic, and chemical interactions. Protein Sci. 30, 187–200. doi: 10.1002/pro.3978, PMID: 33070389 PMC7737760

[ref67] Pechstein A. Bacetic J. Vahedi-Faridi A. Gromova K. Sundborger A. Tomlin N.. (2010). Regulation of synaptic vesicle recycling by complex formation between intersectin 1 and the clathrin adaptor complex AP2. Proc. Natl. Acad. Sci. USA 107, 4206–4211. doi: 10.1073/pnas.0911073107, PMID: 20160082 PMC2840162

[ref68] Pei Y. A. Davies J. Zhang M. Zhang H. T. (2020). The role of synaptic dysfunction in Alzheimer's disease. J. Alzheimers Dis. 76, 49–62. doi: 10.3233/JAD-191334, PMID: 32417776

[ref69] Raj T. Li Y. I. Wong G. Humphrey J. Wang M. Ramdhani S.. (2018). Integrative transcriptome analyses of the aging brain implicate altered splicing in Alzheimer’s disease susceptibility. Nat. Genet. 50, 1584–1592. doi: 10.1038/s41588-018-0238-1, PMID: 30297968 PMC6354244

[ref70] Rajan K. B. Weuve J. Barnes L. L. Mcaninch E. A. Wilson R. S. Evans D. A. (2021). Population estimate of people with clinical Alzheimer's disease and mild cognitive impairment in the United States (2020–2060). Alzheimer Dementia 17, 1966–1975. doi: 10.1002/alz.12362, PMID: 34043283 PMC9013315

[ref71] Sakaba T. Kononenko N. L. Bacetic J. Pechstein A. Schmoranzer J. Yao L.. (2013). Fast neurotransmitter release regulated by the endocytic scaffold intersectin. Proc. Natl. Acad. Sci. USA 110, 8266–8271. doi: 10.1073/pnas.1219234110, PMID: 23633571 PMC3657817

[ref72] Sanchez-Mico M. V. Jimenez S. Gomez-Arboledas A. Muñoz-Castro C. Romero-Molina C. Navarro V.. (2021). Amyloid-β impairs the phagocytosis of dystrophic synapses by astrocytes in Alzheimer's disease. Glia 69, 997–1011. doi: 10.1002/glia.23943, PMID: 33283891

[ref73] Sandau U. S. Duggan E. Shi X. Smith S. J. Huckans M. Schutzer W. E.. (2020a). Methamphetamine use alters human plasma extracellular vesicles and their microRNA cargo: an exploratory study. J. Extracell. Ves. 10:e12028. doi: 10.1002/jev2.12028 PMC7890470 33613872

[ref74] Sandau U. S. Wiedrick J. T. Smith S. J. Mcfarland T. J. Lusardi T. A. Lind B.. (2020b). Performance of validated MicroRNA biomarkers for Alzheimer’s disease in mild cognitive impairment. J. Alzheimers Dis. 78, 245–263. doi: 10.3233/JAD-200396 32955460 PMC9262405

[ref75] Schreij A. M. A. Fon E. A. Mcpherson P. S. (2016). Endocytic membrane trafficking and neurodegenerative disease. Cell. Mol. Life Sci. 73, 1529–1545. doi: 10.1007/s00018-015-2105-x, PMID: 26721251 PMC11108351

[ref76] Selkoe D. J. Hardy J. (2016). The amyloid hypothesis of Alzheimer's disease at 25 years. EMBO Mol. Med. 8, 595–608. doi: 10.15252/emmm.201606210, PMID: 27025652 PMC4888851

[ref77] Sengar A. S. (1999). The EH and SH3 domain Ese proteins regulate endocytosis by linking dynamin and Eps15. EMBO J. 18, 1159–1171. 10064583 10.1093/emboj/18.5.1159 PMC1171207

[ref78] Shim H. S. Horner J. W. Wu C. J. Li J. Lan Z. D. Jiang S.. (2021). Telomerase reverse transcriptase preserves neuron survival and cognition in Alzheimer's disease models. Nat. Aging 1, 1162–1174. doi: 10.1038/s43587-021-00146-z, PMID: 35036927 PMC8759755

[ref79] Shyu A.-B. Wilkinson M. F. Van Hoof A. (2008). Messenger RNA regulation: to translate or to degrade. EMBO J. 27, 471–481. doi: 10.1038/sj.emboj.7601977, PMID: 18256698 PMC2241649

[ref80] Simpson F. Hussain N. K. Qualmann B. Kelly R. B. Kay B. K. Mcpherson P. S.. (1999). SH3-domain-containing proteins function at distinct steps in clathrin-coated vesicle formation. Nat. Cell Biol. 1, 119–124. doi: 10.1038/10091, PMID: 10559884

[ref81] Snyder E. M. Nong Y. Almeida C. G. Paul S. Moran T. Choi E. Y.. (2005). Regulation of NMDA receptor trafficking by amyloid-β. Nat. Neurosci. 8, 1051–1058. doi: 10.1038/nn1503, PMID: 16025111

[ref82] Sormunen A. Koivulehto E. Alitalo K. Saksela K. Laham-Karam N. Ylä-Herttuala S. (2023). Comparison of automated and traditional Western blotting methods. Methods Protoc. 6:43. doi: 10.3390/mps6020043, PMID: 37104025 PMC10142486

[ref83] Srinivasan S. Gal J. Bachstetter A. Nelson P. T. (2022). Alpha adaptins show isoform-specific association with neurofibrillary tangles in Alzheimer's disease. Neuropathol. Appl. Neurobiol. 48:e12776. doi: 10.1111/nan.12776, PMID: 34820873 PMC8810620

[ref84] Stenmark H. Parton R. G. Steele-Mortimer O. Lütcke A. Gruenberg J. Zerial M. (1994). Inhibition of rab5 GTPase activity stimulates membrane fusion in endocytosis. EMBO J. 13, 1287–1296. doi: 10.1002/j.1460-2075.1994.tb06381.x, PMID: 8137813 PMC394944

[ref85] Szabo M. P. Mishra S. Knupp A. Young J. E. (2022). The role of Alzheimer's disease risk genes in endolysosomal pathways. Neurobiol. Dis. 162:105576. doi: 10.1016/j.nbd.2021.105576, PMID: 34871734 PMC9071255

[ref86] Teerlink C. C. Miller J. B. Vance E. L. Staley L. A. Stevens J. Tavana J. P.. (2021). Analysis of high-risk pedigrees identifies 11 candidate variants for Alzheimer's disease. Alzheimers Dement. 18, 307–317. doi: 10.1002/alz.12397 34151536 PMC9291865

[ref87] Terry R. D. Masliah E. Salmon D. P. Butters N. Deteresa R. Hill R.. (1991). Physical basis of cognitive alterations in Alzheimer's disease: synapse loss is the major correlate of cognitive impairment. Ann. Neurol. 30, 572–580. doi: 10.1002/ana.410300410, PMID: 1789684

[ref88] Thomas S. Ritter B. Verbich D. Sanson C. Bourbonnière L. Mckinney R. A.. (2009). Intersectin regulates dendritic spine development and Somatodendritic endocytosis but not synaptic vesicle recycling in hippocampal neurons. J. Biol. Chem. 284, 12410–12419. doi: 10.1074/jbc.M809746200, PMID: 19258322 PMC2673308

[ref89] Tierney M. C. Fisher R. H. Lewis A. J. Zorzitto M. L. Snow W. G. Reid D. W.. (1988). The NINCDS-ADRDA work group criteria for the clinical diagnosis of probable Alzheimer's disease. A clinicopathologic study of 57 cases. Neurology 38:359. doi: 10.1212/wnl.38.3.359 3347338

[ref90] Tong X. K. Hussain N. K. Adams A. G. O’Bryan J. P. Mcpherson P. S. (2000). Intersectin can regulate the Ras/MAP kinase pathway independent of its role in endocytosis. J. Biol. Chem. 275, 29894–29899. doi: 10.1074/jbc.M004096200, PMID: 10896662

[ref91] Tsyba L. Gryaznova T. Dergai O. Dergai M. Skrypkina I. Kropyvko S.. (2008). Alternative splicing affecting the SH3A domain controls the binding properties of intersectin 1 in neurons. Biochem. Biophys. Res. Commun. 372, 929–934. doi: 10.1016/j.bbrc.2008.05.156, PMID: 18539136

[ref92] Tsyba L. Nikolaienko O. Dergai O. Dergai M. Novokhatska O. Skrypkina I.. (2011). Intersectin multidomain adaptor proteins: regulation of functional diversity. Gene 473, 67–75. doi: 10.1016/j.gene.2010.11.016, PMID: 21145950

[ref93] Wiedrick J. T. Phillips J. I. Lusardi T. A. Mcfarland T. J. Lind B. Sandau U. S.. (2019). Validation of MicroRNA biomarkers for Alzheimer's disease in human cerebrospinal fluid. J. Alzheimers Dis. 67, 875–891. doi: 10.3233/JAD-180539, PMID: 30689565 PMC6687305

[ref94] Wilmot B. Mcweeney S. K. Nixon R. R. Montine T. J. Laut J. Harrington C. A.. (2008). Translational gene mapping of cognitive decline. Neurobiol. Aging 29, 524–541. doi: 10.1016/j.neurobiolaging.2006.11.008 17174450 PMC2684335

[ref95] Wong K. A. Wilson J. Russo A. Wang L. Okur M. N. Wang X.. (2012). Intersectin (ITSN) family of scaffolds function as molecular hubs in protein interaction. Networks 7:e36023. doi: 10.1371/journal.pone.0036023 PMC3338775 22558309

[ref96] Wu F. Yao P. J. (2009). Clathrin-mediated endocytosis and Alzheimer's disease: an update. Ageing Res. Rev. 8, 147–149. doi: 10.1016/j.arr.2009.03.002, PMID: 19491039

[ref97] Yao P. J. Morsch R. Callahan L. M. Coleman P. D. (1999). Changes in synaptic expression of clathrin assembly protein AP180 in Alzheimer's disease analysed by immunohistochemistry. Neuroscience 94, 389–394. doi: 10.1016/S0306-4522(99)00360-7, PMID: 10579202

[ref98] Yao P. J. Weimer J. M. O'herron T. M. Coleman P. D. (2000). Clathrin assembly protein AP-2 is detected in both neurons and glia, and its reduction is prominent in layer II of frontal cortex in Alzheimer's disease. Neurobiol. Aging 21, 921–929. doi: 10.1016/S0197-4580(00)00228-1, PMID: 11124443

[ref99] Yao P. J. Zhu M. Pyun E. I. Brooks A. I. Therianos S. Meyers V. E.. (2003). Defects in expression of genes related to synaptic vesicle trafficking in frontal cortex of Alzheimer's disease. Neurobiol. Dis. 12, 97–109. doi: 10.1016/S0969-9961(02)00009-8, PMID: 12667465

[ref100] Yu Y. Chu P.-Y. Bowser D. N. Keating D. J. Dubach D. Harper I.. (2008). Mice deficient for the chromosome 21 ortholog Itsn1 exhibit vesicle-trafficking abnormalities. Hum. Mol. Genet. 17, 3281–3290. doi: 10.1093/hmg/ddn224, PMID: 18676989

