# Supplementary material for: A clathrin mediated endocytosis scaffolding protein, Intersectin 1, changes in an isoform, brain region, and sex specific manner in Alzheimer’s disease
Source: Front Neurosci. 2024 Jun 10;18:1426180. doi: 10.3389/fnins.2024.1426180 (PMC11195150; doi:10.3389/fnins.2024.1426180)
Supplement: Supplementary file 1 [file Data_Sheet_1.PDF]

Table S1. Antibodies Used in This Study. Primary and secondary antibodies used for immunoblots (IB) and immunofluorescence (IF).

Antibody	Host Species	Dilution	Vendor, Product #
ITSN1	Rabbit	IB: 1:2500	Novus Biologicals, NBP1-87806
CLTA	Rabbit	1:2500	Novus Biologicals, NBP2-38638
DNM2	Rabbit	1:1000	Novus Biologicals, NBP2-47477
PICALM	Rabbit	1:1000	Atlas Antibodies, HPA019053
FCHO1	Rabbit	1:200	Invitrogen, PA5-31603
AP2A1	Goat	1:16000	LSBio, LS-B9566
Alpha tubulin	Rat	1:500	Thermo Fisher Scientific, MA1-80017
MAP2	Chicken	1:10000	Thermo Fisher Scientific, PA1-10005
Alexa Fluor 488 Goat anti-Rabbit IgG	Goat	1:500	Thermo Fisher Scientific, A11008
Alexa Fluor 647 Goat anti-Rat IgG	Goat	1:1000	Thermo Fisher Scientific, A21247
Alexa Fluor 647 Donkey anti-Chicken IgY	Donkey	1:500	Thermo Fisher Scientific, A78952
Alexa Fluor 568 Donkey anti-Rabbit IgG	Donkey	1:500	Thermo Fisher Scientific, A10042
IRDye 800CW Donkey anti-Rabbit	Donkey	1:20000	LI-COR Biosciences, 926-32213
IRDye 680RD Donkey anti-Mouse	Donkey	1:20000	LI-COR Biosciences, 926-68072

Table S2. Statistics for Frontal Cortex Immunoblot Results. This table shows the 2-way ANOVA results for frontal cortex (FC) immunoblots. Statistically significant results are in bold and trends are italicized.

	**ITSN1-L: Total Protein**		**2-way ANOVA**			**Post-Hoc tests**	
**Frontal Cortex**	**Group**	**Mean ± SD**		**F(DFn, DFd)**	**p-value**	**Šídák's multiple comparisons test**	
ITSN1-L 190 kDa	Male CTL	50 ± 28	Interaction	F (1, 40) = 0.792	P=0.379	Male CTL vs AD	**P=0.049**
	Male AD	29 ± 20	Sex	F (1, 40) = 3.718	*P=0.061*	Female CTL vs AD	P=0.493
	Female CTL	32 ± 14	Diagnosis	F (1, 40) = 5.820	**P=0.021**	CTL Male vs Female	P=0.103
	Female AD	22 ± 16				AD Male vs Female	P=0.716
CLTCa	Male CTL	1817 ± 79	Interaction	F (1, 39) = 6.652	**P=0.014**	Male CTL vs AD	*P=0.055*
	Male AD	1046 ± 916	Sex	F (1, 39) = 9.223	**P=0.004**	Female CTL vs AD	P=0.323
	Female CTL	458 ± 370	Diagnosis	F (1, 39) = 0.368	P=0.548	CTL Male vs Female	**P=0.0007**
	Female AD	935 ± 880				AD Male vs Female	P=0.935
CLTCb	Male CTL	1967 ± 552	Interaction	F (1, 41) = 0.384	P=0.539	Male CTL vs AD	P=0.253
	Male AD	1523 ± 809	Sex	F (1, 41) = 6.012	**P=0.019**	Female CTL vs AD	P=0.753
	Female CTL	1342 ± 689	Diagnosis	F (1, 41) = 2.439	P=0.126	CTL Male vs Female	*P=0.066*
	Female AD	1151 ± 673				AD Male vs Female	P=0.373
PICALM 65-75 kDa	Male CTL	3409 ± 1491	Interaction	F (1, 41) = 0.829	P=0.368	–	–
	Male AD	3543 ± 1113	Sex	F (1, 41) = 1.185	P=0.283		
	Female CTL	3330 ± 1654	Diagnosis	F (1, 41) = 0.372	P=0.545		
	Female AD	2652 ± 1596					
PICALM 50 kDa	Male CTL	242 ± 53	Interaction	F (1, 39) = 2.589	P=0.116	–	–
	Male AD	430 ± 235	Sex	F (1, 39) = 0.0002	P=0.989		
	Female CTL	341 ± 147	Diagnosis	F (1, 39) = 2.232	P=0.143		
	Female AD	334 ± 270					
AP2A1	Male CTL	58 ± 25	Interaction	F (1, 40) = 2.487	P=0.123	–	–
	Male AD	59 ± 18	Sex	F (1, 40) = 0.182	P=0.672		
	Female CTL	70 ± 14	Diagnosis	F (1, 40) = 1.920	P=0.174		
	Female AD	51 ± 24					
FCHO1	Male CTL	52 ± 21	Interaction	F (1, 40) = 0.010	P=0.922	–	–
	Male AD	44 ± 14	Sex	F (1, 40) = 1.384	P=0.246		
	Female CTL	46 ± 20	Diagnosis	F (1, 40) = 2.165	P=0.149		
	Female AD	37 ± 16					
DNM2	Male CTL	295 ± 174	Interaction	F (1, 41) = 0.0637	P=0.802	–	–
	Male AD	286 ± 124	Sex	F (1, 41) = 0.0890	P=0.767		
	Female CTL	293 ± 118	Diagnosis	F (1, 41) = 0.222	P=0.640		
	Female AD	263 ± 122					

Table S3. Statistics for Hippocampus Immunoblot Results. This table shows the 2-way ANOVA results for hippocampal immunoblots. Statistically significant results are in bold and trends are italicized.

	ITSN1-L: Total Protein		2-way ANOVA			Post-Hoc tests	
**Hippocampus**	**Group**	**Mean ± SD**		**F(DFn, DFd)**	**p-value**	**Šídák multiple comparisons test**	
ITSN1-L 190 kDa	Male CTL	13 ± 12	Interaction	F (1, 52) = 6.45	**P=0.014**	Male CTL vs AD	P=0.459
	Male AD	23 ± 16	Sex	F (1, 52) = 9.372	**P=0.004**	Female CTL vs AD	**P=0.034**
	Female CTL	49 ± 43	Diagnosis	F (1, 52) = 0.890	P=0.287	CTL Male vs Female	**P=0.001**
	Female AD	26 ± 18				AD Male vs Female	P=0.897
ITSN1-S 130 kDa	Male CTL	40 ± 33	Interaction	F (1, 52) = 0.025	P=0.875	Male CTL vs AD	*P=0.053*
	Male AD	80 ± 50	Sex	F (1, 52) = 0.104	P=0.749	Female CTL vs AD	**P=0.031**
	Female CTL	41 ± 31	Diagnosis	F (1, 52) = 11.420	**P=0.001**	CTL Male vs Female	P=0.993
	Female AD	86 ± 56				AD Male vs Female	P=0.912
CLTCa	Male CTL	279 ± 187	Interaction	F (1, 49) = 0.001	P=0.972	–	–
	Male AD	332 ± 152	Sex	F (1, 49) = 0.505	P=0.481		
	Female CTL	316 ± 151	Diagnosis	F (1, 49) = 1.025	P=0.316		
	Female AD	366 ± 202					
CLTCb	Male CTL	289 ± 115	Interaction	F (1, 51) = 0.874	P=0.354	–	–
	Male AD	358 ± 107	Sex	F (1, 51) = 3.429	P=0.070		
	Female CTL	422 ± 257	Diagnosis	F (1, 51) = 0.253	P=0.617		
	Female AD	401 ± 186					
PICALM 65-75 kDa	Male CTL	980 ± 543	Interaction	F (1, 52) = 1.825	P=0.183	–	–
	Male AD	1097 ± 427	Sex	F (1, 52) = 1.517	P=0.224		
	Female CTL	1292 ± 378	Diagnosis	F (1, 52) = 0.1412	P=0.709		
	Female AD	1083 ± 433					
PICALM 50 kDa	Male CTL	172 ± 96	Interaction	F (1, 52) = 3.981	*P=0.051*	–	–
	Male AD	208 ± 72	Sex	F (1, 52) = 1.496	P=0.227		
	Female CTL	265 ± 159	Diagnosis	F (1, 52) = 0.553	P=0.461		
	Female AD	186 ± 94					
AP2A1	Male CTL	42 ± 33	Interaction	F (1, 51) = 0.619	P=0.435	–	–
	Male AD	49 ± 15	Sex	F (1, 51) = 1.999	P=0.163		
	Female CTL	55 ± 26	Diagnosis	F (1, 51) = 0.112	P=0.740		
	Female AD	52 ± 14					
FCHO1	Male CTL	93 ± 37	Interaction	F (1, 50) = 0.0853	P=0.772	–	–
	Male AD	83 ± 40	Sex	F (1, 50) = 0.0219	P=0.883		
	Female CTL	98 ± 49	Diagnosis	F (1, 50) = 1.487	P=0.229		
	Female AD	81 ± 36					
DNM2	Male CTL	53 ± 21	Interaction	F (1, 51) = 1.042	P=0.312	–	–
	Male AD	80 ± 33	Sex	F (1, 51) = 3.517	*P=0.066*		
	Female CTL	79 ± 45	Diagnosis	F (1, 51) = 3.817	*P=0.056*		
	Female AD	87 ± 28					

Table S4. Statistics for Cortical and Hippocampal ITSN1 Immunoblot Results. This table shows the 2-way ANOVA results for hippocampal and cortex immunoblots of ITSN1-L in 5xFAD and WT mice over time. Statistically significant results are in bold and trends italicized.

	ITSN1-L : Total Protein		2-way ANOVA results			Post-Hoc tests	
**Cortex**	**Group**	**Mean ± SD**		**F (DFn, DFd)**	**P value**	**Šídák multiple comparisons test**	
1 month	Male WT	402 ± 33	Interaction	F (1, 23) = 0.299	P=0.590	–	–
	Male 5X	412 ± 75	Sex	F (1, 23) = 0.910	P=0.350		
	Female WT	413 ± 78	Genotype	F (1, 23) = 0.846	P=0.367		
	Female 5X	450 ± 70					
3 months	Male WT	446 ± 68	Interaction	F (1, 19) = 0.909	P=0.353	Male WT vs 5X	P=0.324
	Male 5X	507 ± 98	Sex	F (1, 19) = 1.341	P=0.261	Female WT vs 5X	**P=0.031**
	Female WT	379 ± 51	Genotype	F (1, 19) = 8.320	**P=0.0095**	WT Male vs Female	P=0.265
	Female 5X	501 ± 78				5X Male vs Female	P=0.988
6 months	Male WT	460 ± 112	Interaction	F (1, 22) = 3.550	P=0.0728	Male WT vs 5X	P=0.911
	Male 5X	440 ± 112	Sex	F (1, 22) = 0.3573	P=0.5561	Female WT vs 5X	**P=0.011**
	Female WT	548 ± 44	Genotype	F (1, 22) = 5.958	**P=0.023**	WT Male vs Female	P=0.200
	Female 5X	394 ± 71				5X Male vs Female	P=0.582
9 months	Male WT	844 ± 85	Interaction	F (1, 22) = 0.668	P=0.423	Male WT vs 5X	**P=0.001**
	Male 5X	525 ± 191	Sex	F (1, 22) = 0.080	P=0.780	Female WT vs 5X	**P=0.040**
	Female WT	771 ± 139	Genotype	F (1, 22) = 16.00	**P=0.0006**	WT Male vs Female	P=0.722
	Female 5X	561 ± 185				5X Male vs Female	P=0.903
**Hippocampus**							
1 month	Male WT	281 ± 49	Interaction	F (1, 21) = 0.326	P=0.574	–	–
	Male 5X	292 ± 54	Sex	F (1, 21) = 0.006	P=0.937		
	Female WT	272 ± 34	Genotype	F (1, 21) = 1.339	P=0.2603		
	Female 5X	304 ± 14					
3 months	Male WT	621 ± 96	Interaction	F (1, 18) = 1.053	P=0.318	–	–
	Male 5X	601 ± 27	Sex	F (1, 18) = 1.217	P=0.285		
	Female WT	564 ± 58	Genotype	F (1, 18) = 0.080	P=0.781		
	Female 5X	599 ± 32					
6 months	Male WT	407 ± 59	Interaction	F (1, 22) = 4.901	**P=0.038**	Male WT vs 5X	**P=0.0002**
	Male 5X	287 ± 40	Sex	F (1, 22) = 1.368	P=0.255	Female WT vs 5X	P=0.222
	Female WT	346 ± 25	Genotype	F (1, 22) = 20.13	**P=0.0002**	WT Male vs Female	*P=0.061*
	Female 5X	305 ± 54				5X Male vs Female	P=0.697
9 months	Male WT	406 ± 118	Interaction	F (1, 22) = 1.401	P=0.249	Male WT vs 5X	P=0.286
	Male 5X	313 ± 35	Sex	F (1, 22) = 4.702	**P=0.041**	Female WT vs 5X	**P=0.004**
	Female WT	547 ± 129	Genotype	F (1, 22) = 11.71	**P=0.0024**	WT Male vs Female	*P=0.057*
	Female 5X	354 ± 103				5X Male vs Female	P=0.738

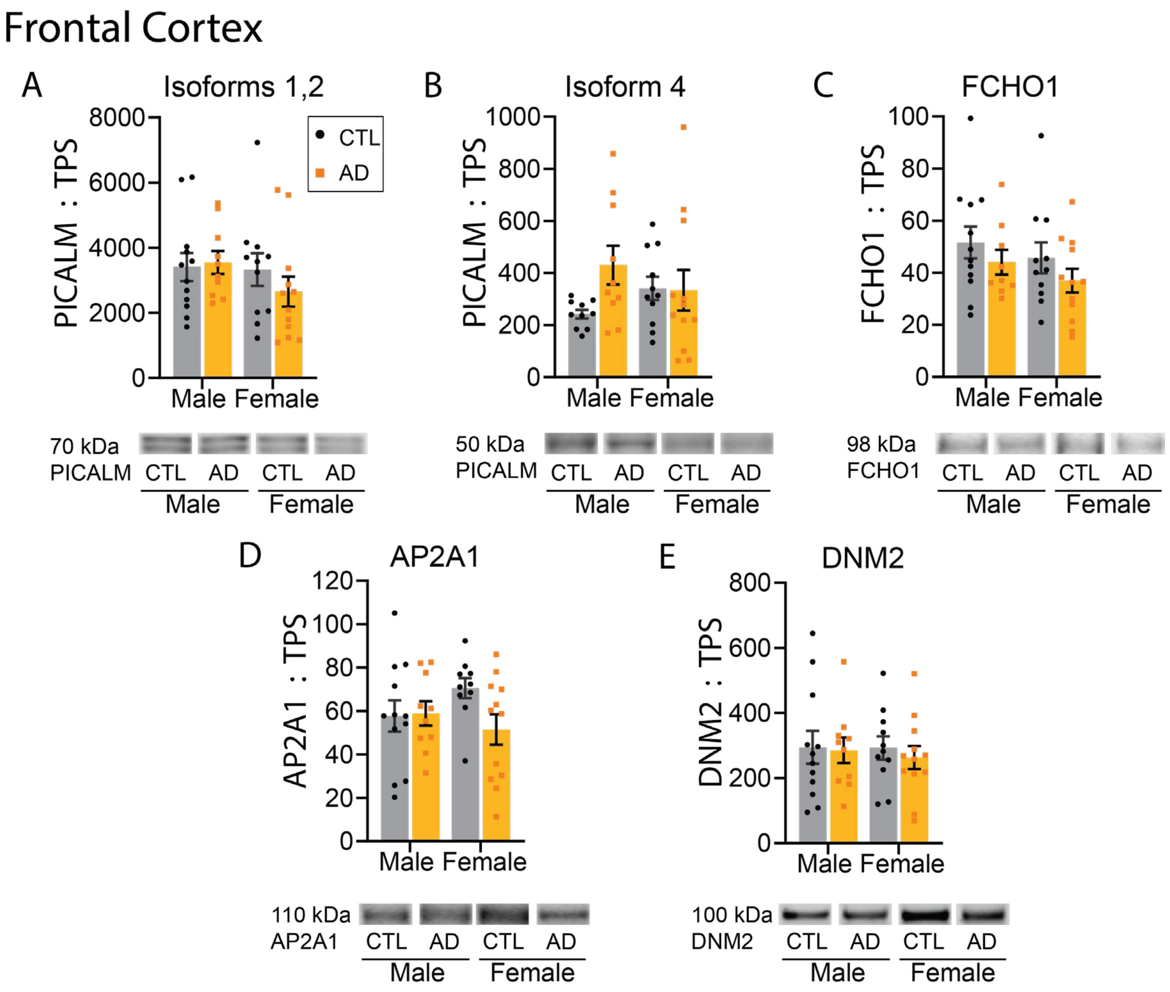

Figure S1. Other CME Proteins Are Not Changed in AD Frontal Cortex. Comparison of proteins involved in CME between males and females in AD and CTL frontal cortex (FC). Proteins examined include PICALM isoforms 1,2 (A) and isoform 4 (B), FCHO1 (C), AP2A1 (D), and DNM2 (E). For each analysis, all graphs show protein intensity values normalized to total protein stain (TPS) with representative immunoblot bands underneath. Data are represented as mean ± SEM. Statistical significance was determined by two-way ANOVA, with post-hoc Šídák multiple comparisons tests for analyses with significant group effects. *P<0.05

Hippocampus

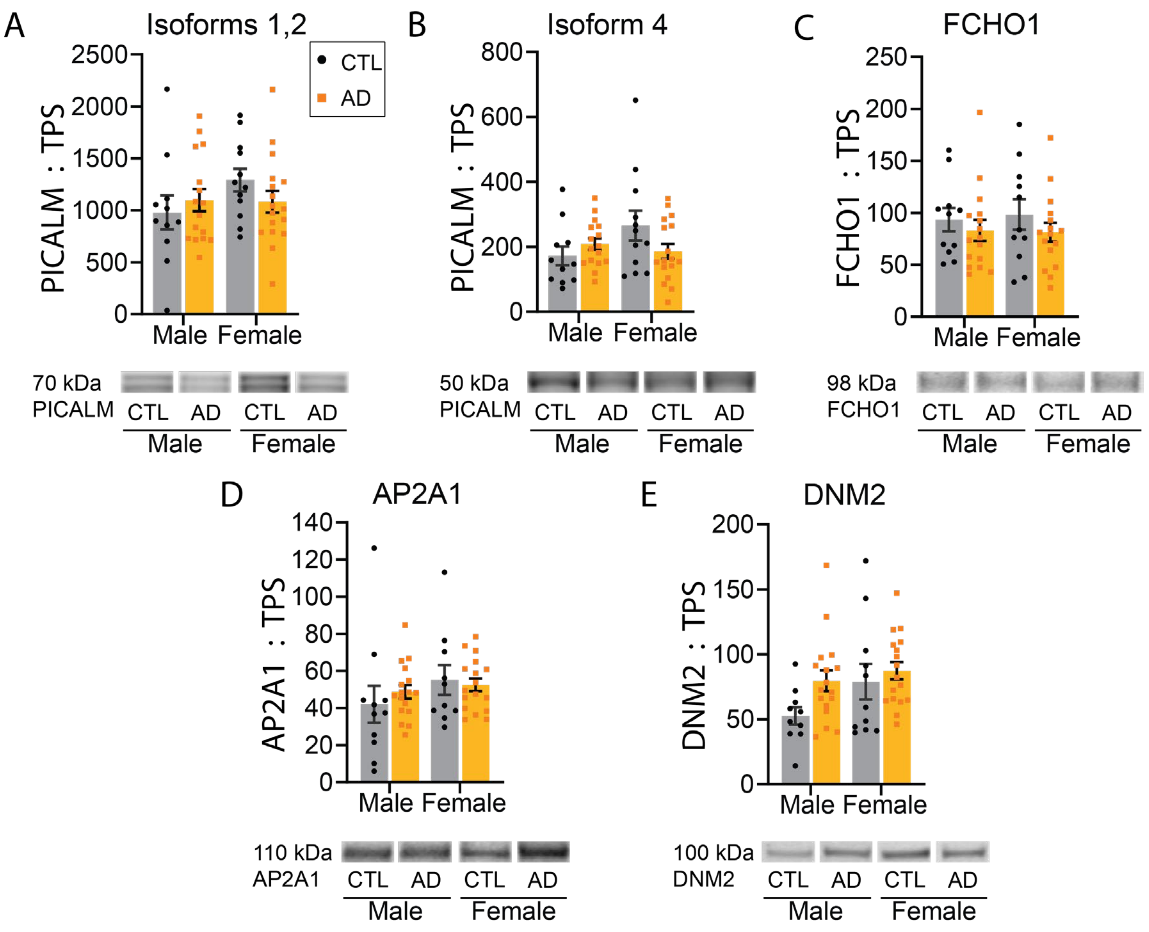

Figure S2. **Other CME Proteins Are Not Changed in AD Hippocampus.** Comparison of proteins involved in CME between males and females in AD and CTL hippocampus. Proteins examined include PICALM isoforms 1,2 (A) and isoform 4 (B), FCHO1 (C), AP2A1 (D), and DNM2 (E). For each analysis, all graphs show protein intensity values normalized to total protein stain (TPS) with representative immunoblot bands underneath. Data are represented as mean ± SEM. Statistical significance was determined by two-way ANOVA, with post-hoc Šídák multiple comparisons tests for analyses with significant group effects. *P<0.05

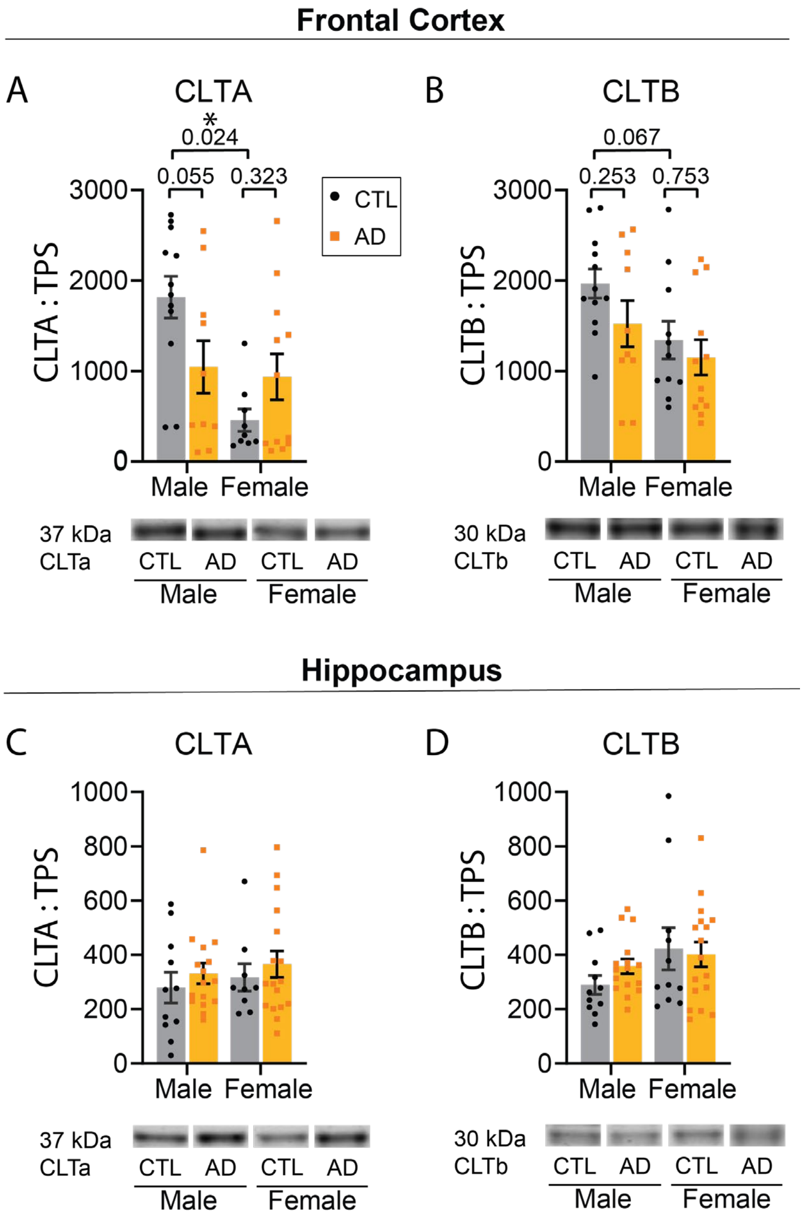

Figure S3. Clathrin Light Chain Shows No Change In AD but Reveals a Significant Sex Difference in CTLs. Comparison of Clathrin Light Chain A (CLTA) and Clathrin Light Chain B (CLTB) between males and females in AD and CTL frontal cortex (FC) and hippocampus (HP). For each analysis, all graphs (A, B, C, D) show protein intensity values normalized to total protein stain (TPS) with representative immunoblot bands underneath. Data are represented as mean ± SEM. Statistical significance was determined by two-way ANOVA, with post-hoc Šídák multiple comparisons tests for analyses with significant group effects. *P<0.05

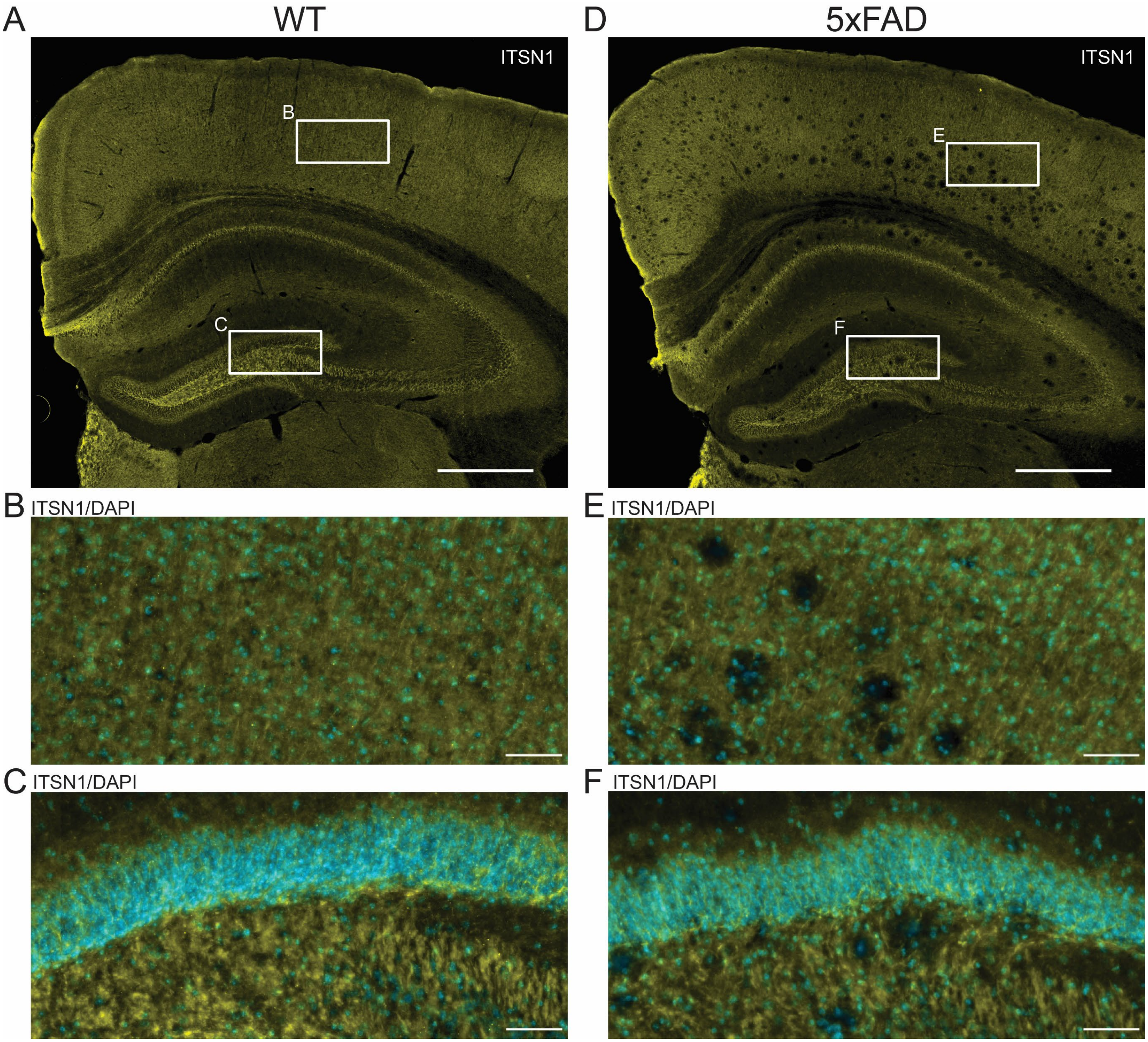

Figure S4. ITSN1 Expression Visualized in Female 9-month Wildtype and 5xFAD Mice. Representative wide field images of female 9 month old WT (A) and 5xFAD (B) mouse brains stained for ITSN1 (yellow). White boxes in indicate areas magnified in the cortex (B) and hippocampus (C) in WT, and the cortex (E) and hippocampus (F) in 5xFAD. Each region is shown with both ITSN1 (yellow) and DAPI (cyan). Scale bars are 500 μm in A and D, and 50 μm in C, D, and E.
